# An RNA origami robot that traps and releases a fluorescent aptamer

**DOI:** 10.1126/sciadv.adk1250

**Published:** 2024-03-20

**Authors:** Néstor Sampedro Vallina, Ewan K. S. McRae, Cody Geary, Ebbe S. Andersen

**Affiliations:** ^1^ Interdisciplinary Nanoscience Center, Aarhus University, Aarhus, Denmark.; ^2^ Center for RNA Therapeutics, Department of Cardiovascular Sciences, Houston Methodist Research Institute, 6670 Bertner Ave, R10-117, Houston, TX 77030, USA.; ^3^ Department of Molecular Biology and Genetics, Aarhus University, Aarhus, Denmark.

## Abstract

RNA nanotechnology aims to use RNA as a programmable material to create self-assembling nanodevices for application in medicine and synthetic biology. The main challenge is to develop advanced RNA robotic devices that both sense, compute, and actuate to obtain enhanced control over molecular processes. Here, we use the RNA origami method to prototype an RNA robotic device, named the “Traptamer,” that mechanically traps the fluorescent aptamer, iSpinach. The Traptamer is shown to sense two RNA key strands, acts as a Boolean AND gate, and reversibly controls the fluorescence of the iSpinach aptamer. Cryo–electron microscopy of the closed Traptamer structure at 5.45-angstrom resolution reveals the mechanical mode of distortion of the iSpinach motif. Our study suggests a general approach to distorting RNA motifs and a path forward to build sophisticated RNA machines that through sensing, computing, and actuation modules can be used to precisely control RNA functionalities in cellular systems.

## Teaser

The RNA origami method was used to construct robotic devices that mechanically trap and release RNA functional motifs.

## INTRODUCTION

DNA nanotechnology exploits the regular structure of DNA double helices to construct higher-order three-dimensional (3D) structures and the simple base pairing rules to design sequences that self-assemble into these structures (*1*). To make the nanostructures dynamic, the toehold-mediated strand displacement (TMSD) mechanism is often used to reconfigure base pairings to create molecular machines (*2*, *3*) or logic circuits (*4*–*6*). The introduction of the DNA origami method laid the basis for designing increasingly complex DNA nanostructures by heat-annealing a long scaffold strand with hundreds of smaller staple strands (*7*). DNA origami allowed the construction of molecular machines, such as nanocontainers, that can be opened by sequence-specific key signals (*8*, *9*). We refer to these as “nanorobots” since they are nanomechanical devices that can sense inputs, do basic computations, and act within their nanoscale environment. Several examples of these robotic devices have been developed to solve tasks of targeting and killing cancer cells (*10*), delivering immunostimulatory cargoes to dendritic cells (*11*), and delivering blood clotting agents to tumors (*12*).

RNA nanotechnology exploits the wider variety of structural and functional motifs found in natural RNAs (*13*). The tertiary RNA motifs are formed by both canonical and noncanonical base pairs, resulting in more complex base pairing rules and sequences that are more difficult to design (*14*, *15*). Several RNA nanoparticles have been developed by the tecto method and used for a variety of applications (*16*–*20*). We developed the RNA origami method by combining crossover motifs derived from DNA nanotechnology with tertiary loop motifs from RNA nanotechnology (*21*). The RNA origami structures are made from a single strand and can self-assemble through cotranscriptional folding, an important feature enabling genetic encoding and expression in cells (*22*). Software for RNA origami facilitates the design of large structures with the addition of functional motifs (*23*), primarily used for creating functional scaffolds in RNA medicine (*24*, *25*) and RNA synthetic biology (*26*, *27*). In addition, RNA origami has been used in the creation of dynamic devices (*22*) and has been observed to form kinetic traps that mature over time (*28*) but has not yet been used for implementing robotic devices capable of sensing, computing, and actuating.

Inspiration for designing RNA devices can be drawn from the mechanisms of riboswitches (*29*–*31*) which typically function through ligand binding, inducing a conformational change that releases a specific functional motif (*32*, *33*). Inspired by these mechanisms, researchers have integrated target-binding aptamers with functional sequences to control activities such as splicing (*34*), transcription (*35*), and translation (*36*, *37*). Alternatively, toehold switches have been used, which activate gene expression through strand displacement (*37*). By combining more RNA devices, Boolean logic can be implemented to activate or deactivate biological functions (*36*–*39*). The development of fluorescent aptamer technology has provided RNA devices with a fluorescence output signal (*40*, *41*), facilitating the creation of several RNA sensors for diverse ligands by linking ligand-binding aptamers with fluorescent aptamers through a transducer module (*42*–*45*). In addition, to control fluorescent aptamers, TMSD can be used (*46*–*49*). In the examples of riboswitches and TMSD devices mentioned above, the transduction mechanism operates through base pair reconfiguration. However, the exploration of RNA devices using nanomechanical transduction and computation to regulate functional RNA motifs has yet to be fully explored.

Here, we use the RNA origami method to develop an RNA robotic device, named the “Traptamer.” This device can sense two RNA key strands, function as a Boolean AND gate, and trigger the activation of the iSpinach fluorescent aptamer by releasing it from a mechanical trap. To obtain this complex device, we screen several designs for the ability to trap the iSpinach aptamer in an inactive state and to release it to fold in its native conformation, binding its cognate fluorogenic dye. Upon identifying a device with the desired properties, we investigate its ability to reversibly open and close. Last, we use cryo–electron microscopy (cryo-EM) to determine the structure of the closed form of the Traptamer at 5.45-Å resolution, revealing details of the mechanical distortion of the iSpinach motif. The Traptamer exemplifies a general approach to controlling RNA functionality, which holds promise for a wide range of applications in RNA medicine and synthetic biology.

## RESULTS

### Design of an RNA origami trap for aptamers

RNA origami structures are composed of multiple helices that are connected by double crossovers, where the distance between crossovers corresponds to an integer number of full turns of the A-form helix of 11 base pairs (bp) per turn (*21*, *23*). However, by making some helices longer and others shorter, we can introduce out-of-plane bending in the longer helices. Our core idea is that placing a functional RNA motif in the longer helix may cause the motif to bend out of shape, thereby inactivating its function. Here, we apply this concept to trap an aptamer in an RNA origami frame and refer to this device as a Traptamer. Specifically, we attempt to demonstrate this concept by trapping the iSpinach aptamer, thereby controlling its binding to its cognate fluorogenic dye, (5*Z*)-5-[(3,5-difluoro-4-hydroxyphenyl)methylene]-3,5-dihydro-2-methyl-3-(2,2,2-trifluoroethyl)-4*H*-imidazol-4-one (DFHBI-1T) (*50*).

**Fig. 1.**
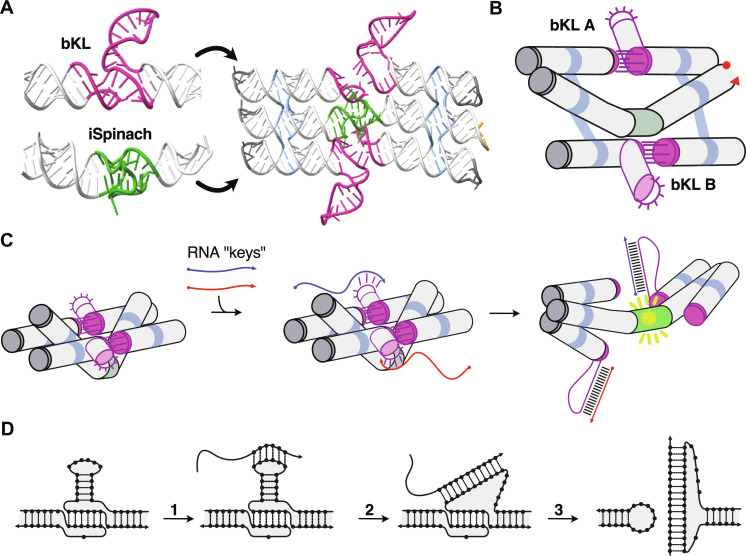
Design of a mechanical trap for aptamers. (**A**) Depiction of the trap design. A three-helix tile RNA origami connected by two double crossovers (blue) has the iSpinach fluorogenic aptamer [Protein Data Bank (PDB): 5OB3] incorporated into the middle helix (green). The structure is bridged together by two branched kissing loops (bKL) (*51*) (magenta), which have an extended 5-bp stem with a 6-nt loop at the end acting as a toehold for strand displacement. (**B**) Schematic representation of the device. (**C**) Schematic representation of the opening mechanism. The strand displacement reaction starts on the 5′ end of the toehold loop of the bKLs, continues along the stem of the bKL, and ends up base pairing along the loop, breaking the kissing interaction; iSpinach is then allowed to fold into its active conformation and bind DFHBI-1T, producing a fluorescent output. (**D**) Schematic representation of the bKL disruption by loop-mediated strand displacement. In step 1, keys bind to the hairpin loop of the bKL; in step 2, the key pairs along the hairpin stem of the bKL; and in step 3, the key breaks the kissing interaction.

To construct the Traptamer, we positioned the iSpinach aptamer in the middle helix of a three-helix RNA origami tile, with the outer helices intentionally kept short to force the iSpinach aptamer out of plane and into a deactivated conformation (Fig. 1, A and B). To enable the trap to open, we integrated branched kissing loops (bKL) (*51*) into the top and bottom helices. The bKLs were extended with a 5-bp stem capped with a 6-nucleotide (nt) loop, which can act as a toehold for strand displacement, as previously demonstrated in (*22*). When the cognate RNA strands, referred to as “RNA keys,” invade the bKLs, the bKL interaction is disrupted, relieving the structural constraints on the central helix. This allows iSpinach to refold and bind DFHBI-1T (Fig. 1C). The proposed mechanism for loop-mediated strand displacement into the bKL is illustrated in Fig. 1D (with a reversible mechanism shown in fig. S1).

### Screening for iSpinach trapping and release

**Fig. 2.**
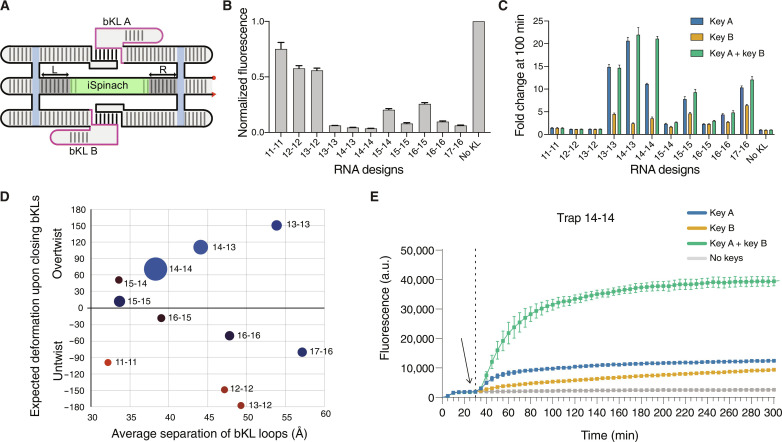
Design screening of RNA origami trapping and release. (**A**) Schematic of the design parameter variations. The length of the double-stranded left (L) and right (R) stems on the middle helix varied from 11 to 17 bp on the L side and from 11 to 16 bp on the R side. (**B**) Background fluorescence observed for the different designs at 100 nM RNA and 500 nM DFHBI-1T before keys were added at 25°C. Data correspond to three technical replicates; error bars represent means ± SD. (**C**) Fold-change fluorescence activation 50 min after the addition of the RNA keys (500 nM) to the different RNA designs (100 nM). Fold change was calculated by comparison to the no-keys sample. Data corresponds to three technical replicates; error bars represent means ± SD. (**D**) Plot of expected twist (in degrees) and bKL separation (in angstrom) in Traptamer designs with data from (B) and (C) shown on the plot: Red = normalized background, blue = normalized response to both keys, size = specificity to both keys over individual keys. (**E**) Observed fluorescence for the 14-14 device over time at 25°C. DFHBI-1T (500 nM) was added after the first measurement; cognate and negative control keys (500 nM) were added after 30 min (dashed line). Data correspond to three technical replicates; error bars represent means ± SD. a.u., arbitrary units.

To identify designs with the desired functionality, we generated a series of designs by varying the stem length from the iSpinach aptamer to the crossover on both sides. Each RNA design was labeled “L-R,” where L and R represent the number of bp in the stems to the left and right of the iSpinach motif, respectively (Fig. 2A and table S1). The different RNA variants were produced by in vitro transcription, purified by size exclusion chromatography, and their fluorescence in complex with DFHBI-1T was measured at 25°C (excitation at 470 nm and emission recorded at 505 nm) (fig. S2, A and B). Clear differences in fluorescence were observed among the series compared to a control in which the bKL interactions were disrupted by tetraloops (No-KL normalized to 1.0). Designs 11-11, 12-12, and 13-12 exhibited high fluorescence (>0.5); 15-14 and 16-15 exhibited medium fluorescence (0.2 to 0.5); and 13-13, 14-13, 14-14, 15-15, 16-16, and 16-17 exhibited low fluorescence (<0.2) (Fig. 2B) indicating varying levels of trapping. The inefficient trapping of 15-14 and 16-15 can be attributed to their tendency to assemble into multimers, as observed by gel analysis (fig. S2A).

Subsequently, we tested the response of these designs to the addition of key A, key B, or both. As expected, designs 11-11, 12-12, and 13-12, which already showed high fluorescence in the absence of keys, did not respond to the addition of keys, indicating that the iSpinach aptamer remains untrapped (Fig. 2C and fig. S2C). Designs 13-13 and 14-13 were fully activated by key A (15- to 22-fold) and only 10 to 30% activated by key B alone (2- to 5-fold), suggesting that bKL A is primarily responsible for disrupting iSpinach in these constructs. Design 14-14 showed 52% activation by key A and 16% activation by key B (4- to 11-fold) but only showed full activation when both RNA inputs were present (22-fold). Thus, 14-14 exhibited cooperative behavior, acting similar to an AND gate, indicating that both bKL A and B were formed and independently inhibited the active form of iSpinach. Designs 15-14 and 16-15, which were only partly trapped, showed weak activation by keys. Designs 15-15, 16-16, and 17-16 exhibited similar activation profiles, with approximately 90% activation by key A and approximately 50% activation by key B, suggesting that the opening of one bKL led to the opening of the other, similar to an OR gate.

To rationalize the observed effects in terms of physical properties, we constructed ideal models of all the Traptamer variants using our RNAbuild software (fig. S3) and measured the distance between cognate KLs for each design as well as the direction and amount of twist required to close the KLs and compared it to our experimental data (Fig. 2D). Our analysis revealed that designs with undertwisting above 90° resulted to high fluorescence and no activation by keys, indicating that iSpinach was not trapped (red dots for 11-11, 12-12, and 13-12). Overtwisting above 60° led to low fluorescence and activation by keys, indicating that the iSpinach motif was trapped (blue dots for 13-13, 14-13, and 14-14). Designs with less twist (60° to −80°) exhibited variable inhibition of fluorescence and low activation by keys (darkened dots for 15-14, 15-15, 16-15, 16-16, and 17-16). Overall, we observed that overtwisting was beneficial for inactivating the iSpinach aptamer, and design 14-14 represented a sweet spot for specificity to both keys.

### Traptamer cooperativity and modularity

The properties of the Traptamer 14-14 were investigated further to understand its mode of action. Initially, we determined the number of keys required to fully open the trap and found that it reached maximum response at 5× concentration of both keys, resulting in 83% fluorescent activation after 30 min (fig. S4, A and B). Next, the impact of K^+^ concentration was tested, as monovalent cations play a crucial role in stabilizing the G-quadruplex (G4) motif in iSpinach (*52*). The fluorescence was measured before and after the addition of 5× concentration of both keys at different KCl concentrations (fig. S4, C and D). We observed that fluorescence gradually increased with increasing KCl concentration from 5 to 100 mM, which can be explained by the effect of K^+^ in the maturation of the iSpinach structure (*53*). At higher KCl concentrations of 150 and 200 mM, fluorescence increased more rapidly upon the addition of keys, suggesting that at high KCl concentrations, iSpinach rapidly adopts its mature conformation for binding to the fluorophore.

We also tested alternative keys C and D to demonstrate the specificity of the response to keys A and B (fig. S5A). By monitoring the system over 9 hours, we found that key B’s fluorescence activation increased gradually, approaching the activation level of key A (fig. S5B). This indicates that the difference in fluorescence activation is caused by the rate of strand displacement and correlates with the differences in the free energy of the branched stem of the bKLs (fig. S5C). In later experiments that were not done as part of a larger experimental series, we observed lower fluorescence activation by keys A and B individually (Fig. 2E and fig. S6), suggesting that the time outside the instrument for adding keys can affect the activation kinetics. Given that both input strands are necessary for full activation, we conclude that Traptamer 14-14 functions as a logic AND gate with a threshold set at 30%.

To determine whether the observed behavior of Traptamer 14-14 was affected by the specific sequence design, we designed and synthesized another RNA sequence, 14-14 v2, with the same structural elements (i.e., bKLs, aptamer sequence, and 5′ and 3′ ends) but a different scaffold sequence (table S1). Both Traptamer 14-14 and 14-14 v2 (fig. S6) exhibited similar responses, indicating that the observed behavior was not sequence-specific but can rather attributed to the structural elements of the Traptamer. To further test the modularity of our design, we incorporated the Broccoli aptamer (*54*) into the scaffold sequence and observed similar behavior once again (fig. S6 and table S1). These results indicate that our design is modular and suggest that different aptamers or functional motifs may be regulated using similar strategies.

Previous studies have shown that the binding aptamer influences the photophysical properties of DFHBI-1T (*54*, *55*), indicating that different RNA structures can stabilize the fluorophore in various configurations, resulting in these changes. Therefore, we investigated whether the distinct fluorescent outputs observed from the opening of 14-14 with different keys could also be attributed to different structural states of the Traptamer. To test this hypothesis, we measured the excitation and emission spectra of DFHBI-1T in complex with 14-14 in the presence of different key combinations. However, no notable difference was detected in the fluorescence spectral outputs (fig. S7). Thus, the changes in fluorescence observed can be attributed to the folding or disruption of the iSpinach aptamer.

### Reversibility of the trapping mechanism

**Fig. 3.**
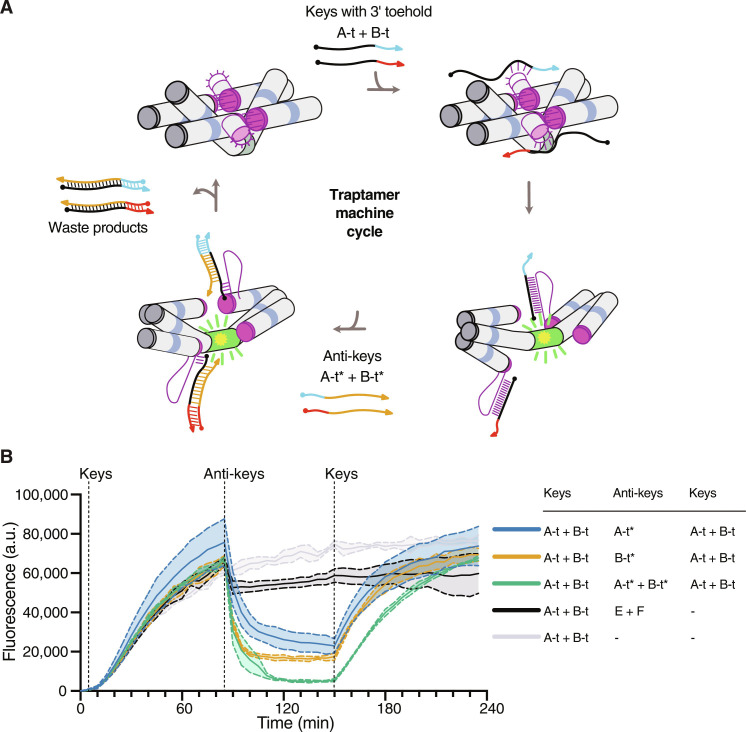
Reversible activation and deactivation of iSpinach by Trap 14-14. (**A**) Schematic representation of the Traptamer machine cycle. The single-stranded RNA keys were extended with 6-nt toeholds (A-t and B-t) and anti-keys were designed to displace the keys (A-t* and B-t*). (**B**) Monitored fluorescence over time of Traptamer 14-14 RNA (100 nM) in solution with DFHBI-1T (500 nM) at 25°C. Keys with toehold (500 nM) were added after 5 min, anti-keys (1 μM) were added after 85 min, and keys (2 μM) were added again after 150 min (at dashed lines) in different combinations according to the table. Data correspond to three technical replicates; error bars represent means ± SD.

The opening of the bKL by a key has previously been shown to be reversible by the addition of an anti-key strand that binds a toehold on the key strand and removes it from the complex by strand displacement (*22*). By adding keys and anti-keys, it may thus be possible to open and close the Traptamer to cycle the aptamer between its on and off state (Fig. 3A). We initially examined the effect of adding a 5-nt toehold to the key strand on either the 5′ or 3′ end, referred to as t-key and key-t, respectively. The keys without toehold activated the fluorescence at a maximum rate of 847 U/min, while t-key activated at a maximum rate of 443 U/min and the key-t activated at a maximum rate of 541 U/min (fig. S8). Although t-key exhibited a delayed response, it reached close to maximum fluorescence after 160 min. In contrast, key-t showed a further delay and stabilized at 60% of maximum fluorescence. The lower efficiency of key-t can be attributed to the placement of two toeholds next to each other, as previous studies have shown that this configuration decreases the strand displacement rates in DNA systems (*56*). Despite reaching lower overall fluorescence, key-t reached its maximum faster, leading us to select it for further experiments.

The reversibility was tested by adding 5× key A-t and key B-t and letting the signal increase for 80 min (Fig. 3B). Subsequently, a 2× excess of either one or both anti-key strands (A-t* and B-t*) was added. This resulted in a sharp decrease in fluorescence, reaching minimum levels after 25 min (Fig. 3B). Both anti-keys can turn off the fluorescent signal, with A-t* being faster than B-t*. The addition of both anti-keys led to complete fluorescence turn-off. When adding a 4× excess of keys after 150 min, we observe a reactivation of the fluorescence that returns to similar levels. The data show that the Traptamer can trap and release the Spinach aptamer in a reversible manner and that the opening appears to be limited by the speed of the strand displacement mechanism. Furthermore, the addition of anti-keys E and F with unrelated sequences does not result in fluorescence turnoff.

### Cryo-EM structure of closed Traptamer

**Fig. 4.**
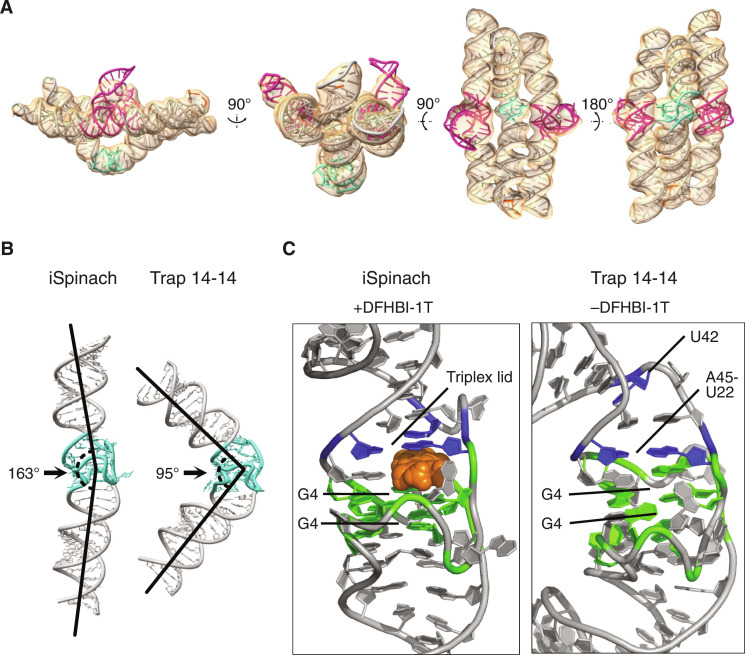
Cryo-EM characterization of the cotranscriptionally assembled state of Traptamer 14-14. (**A**) Cryo-EM map at 5.45-Å resolution (orange) (EMD-41656) with atomic model (PDB: 8TVZ) with bKLs highlighted in magenta, iSpinach in blue, and the rest of the origami in gray. (**B**) Comparison between the bending of the iSpinach aptamer based on the crystal structure (PDB: 5OB3) (left) and the iSpinach aptamer model based on the cryo-EM map (right). (**C**) Comparison of the binding pocket of iSpinach in the crystal structure and the cryo-EM structure shown in cartoon representation. The binding pocket of iSpinach in the Traptamer model appears contorted as compared to the crystal structure. The following elements are colored: G-quadruplex (G4, green), triplex lid (blue), and DFHBI-1T (orange spheres).

The fluorescence data suggest that the formation of the bKLs in design 14-14 disrupts the iSpinach aptamer, preventing it from binding DFHBI-1T. To gain a better understanding of the trapped conformation of the iSpinach aptamer, we used cryo-EM to analyze the cotranscriptionally assembled conformation of 14-14 with and without keys. The reconstruction of the trapped conformation reached a resolution of 5.45 Å (figs. S9 and S10), and the EM map revealed well-resolved double helices and crossovers, as well as the bKLs and a ~90° bend of iSpinach (Fig. 4, A and B). Although we attempted to reconstruct the open state of design 14-14 (i.e., after the addition of the keys) with cryo-EM, the resolution we could attain was limited by the flexibility of the molecule (fig. S11).

To gain a deeper understanding of how the Traptamer disrupts iSpinach, we manually built a model of Traptamer 14-14 into the density map (see Materials and Methods) (Fig. 4A and fig. S10). By flexibly fitting the native iSpinach model into our density map, we obtained a model of the deformed state and its effects on the fluorophore binding pocket (Fig. 4C). In the native state of iSpinach, the fluorophore binding pocket is located between the G4 and an A-U base pair. The cryo-EM model shows that the bending occurs right at the binding pocket, where the A45-U22 base pair occupies the space of the fluorophore. This bending disrupts the triplex lid formed by the A45-U22 base pair and the bulged U42, while the G4s remain generally preserved. The hinge-like deformation provides an explanation for the mechanical deactivation of the iSpinach aptamer inflicted by the forces of bKL formation in the origami frame.

## DISCUSSION

In this work, we have developed an RNA Traptamer device that traps an aptamer in an inactive conformation by embedding it into an origami frame. Using bKLs to lock the frame, the device was designed to recognize small RNA sequences that trigger the opening of the trap and activation of the aptamer. We observe a cooperative effect on fluorescence when adding both keys, which can be interpreted as Boolean AND gate behavior using an appropriate threshold. Furthermore, because the device uses strand displacement for activation, it can also be cycled between on and off states. Thus, we have demonstrated an RNA robotic device that can sense, compute, and actuate by mechanical force in a reversible manner.

The Traptamer 14-14 was found through screening several different designs, wherein the middle helix was expanded in 1- to 2-bp increments. The expected effect of a 1-bp insertion into the middle helix is to increase both the spacing by 2.9 Å and the twist by approximately 32.7°, with the twist being the major factor. By analyzing the twist (Fig. 2D), we found that overtwisting provided the best functioning devices. However, we also observed that the AND-gate behavior of Traptamer 14-14 was lost with the addition or subtraction of just one base pair. Overtwisting of double-stranded RNA has been shown to lead to a decrease in the helical rise and an increase in inclination (*57*), and we therefore hypothesize a mechanism wherein overtwisting of the A-form helices leads to distortion of the iSpinach motif. Since the G4 of the iSpinach motif is very stable, the deformation occurs at the ligand binding site, leading to the ejection of DFHBI-1T and bending at the empty binding site. It has been observed that simultaneous bending and twisting leads to the focusing of forces at a unique breaking point for cylinders, such as dried spaghetti (*58*). In conclusion, we propose overtwisting as a general strategy to distort and bend RNA motifs.

The Traptamer 14-14 activates at a slower rate than standard RNA strand displacement kinetics (*56*). Factors that may contribute to the slow activation kinetics are the following: (i) The energetics of invading a loop are different from invading a free strand since the loop has a lower starting entropy due to being docked at both ends; (ii) steric hindrance of strand displacement into a confined loop; (iii) the high stability of the bKL stems, which was found to correlate with the speed of opening in the presence of keys; (iv) strand displacement into the stable tertiary motif of the kissing loop disrupts highly stable base stacks; and (v) maturation time of the iSpinach aptamer after release from the trap. When testing the reversibility of the Traptamer, we observed a slow activation rate of ~80 min and a fast deactivation rate of ~10 min. The fast off-rate matches the expected time for a strand displacement and, therefore, the formation of kissing loops and inactivation of iSpinach is likely to happen very fast. The slower on-rate and the faster off-rate can be explained by the fact that internal toeholds tend to slow down the strand displacement process (*56*). The speed of activation may be improved by designing less stable bKL stems and by using larger loops to improve toehold binding.

Structural investigation of the 14-14 Traptamer using cryo-EM revealed a bending of the iSpinach aptamer caused by the tension induced on the middle helix due to the formation of the bKLs. Our data suggest that binding sites or active sites may be especially sensitive to mechanical deformation because of the more complex base pairing patterns. This RNA origami framework could be used to study the force required to inactivate the different RNA motifs by changing the number and strength of the kissing loop interactions, effectively tuning the strength of the distortive framework. Just like DNA origami devices are used for measuring forces (*59*–*62*), the RNA origami frames presented here could be used to measure forces. To demonstrate these ideas, we suggest an alternative Traptamer pulling configuration, wherein a single strand replaces part of the middle helix (fig. S12). In the open conformation, the single strand is allowed to fold an aptamer hairpin, which in the closed state is pulled apart by the forces of the bKL-KL formation. The force on the single strand can be increased by extending the RNA origami frame, allowing disruption of G4s and H-type pseudoknots.

Because RNA origami design is highly modular, the Traptamer can be modified to respond to other environmental signals. For example, by using kissing-loop interactions responsive to small molecules (*63*–*65*), the Traptamer could be designed to switch upon sensing other molecules and environmental cues. The modularity of the RNA origami architecture also enables the regulation of different functional RNA motifs using similar strategies. We demonstrated this to a limited extent by testing the incorporation of another reporter aptamer, Broccoli (*54*), into a Traptamer. By incorporating different fluorescent aptamers and bKL sequences in the origami frames, it may be possible to build multiplex sensor platforms for detecting multiple RNA targets simultaneously. Any other functional RNA motifs with similar configurations (i.e., embedded on a stem) such as protein-binding regions could be similarly regulated.

Our study of the Traptamer demonstrates an approach to combine sensing, computing, and actuation modules to obtain molecular devices that rely on allosteric regulation and large conformational changes for their actuation. By combining different modules, a large variety of RNA robotic devices can be constructed for applications in medicine, synthetic biology, and materials science.

## MATERIALS AND METHODS

### Experimental design

The objective of the study was to design an RNA origami trap for aptamers. RNA origami design methods are explained in (*23*). Briefly, the different structural motifs are incorporated into a 2D blueprint using a standard text editor. Sequence constraints were applied at the RNA motifs (bKLs and iSpinach aptamer), the 3′ end with the primer binding site sequence, and the 5′ end with the sequence GGA, an optimal initiation sequence for the T7 RNA polymerase. The remaining unconstrained sequence was then designed using the Perl script “batch-revolvr.pl” available at https://github.com/esa-lab/ROAD and as a web server at https://bion.au.dk/software/rnao-design/. The sequence of the T7 RNA polymerase promoter was introduced upstream of the RNA origami and primers were designed for polymerase chain reaction (PCR) amplification. Blueprints and sequences can be found in table S1.

### Synthesis of DNA templates

The DNA templates for the different RNA designs were produced by PCR amplification of double-stranded gene fragments (gBlocks) synthesized by Integrated DNA Technologies (IDT), as well as the primers (table S2). Amplifications were performed in 100 μl of reactions containing 1× Phusion HF buffer (NEB), 1 μM of each primer, 200 μM deoxyribonucleoside triphosphates (Invitrogen), 4 ng of gBlock template, and 1 U of Phusion High-Fidelity DNA polymerase (NEB). The reaction was subjected to a 2-min initial denaturation at 98°C, followed by 30 cycles of 98°C for 10 s, 68°C for 15 s, and 72°C for 10 s, followed by a final extension step at 72°C for 2 min and cooling down to 10°C. The reaction products were purified using the Macherey-Nagel PCR clean-up kit following the manufacturer’s instructions.

### Production and purification of RNA

RNA was produced by in vitro transcription. Reactions (500 μl) were performed by mixing 3 to 5 μg of the purified DNA templates, 20 mM MgCl_2_, 10 mM ribonucleoside triphosphates (2.5 mM each), 10 mM dithiothreitol, 40 mM Hepes, 50 mM KCl, 2 mM spermidine, and in-house produced T7 RNA polymerase. The reaction was incubated at 37°C for 3 hours and stopped by adding 2 U of deoxyribonuclease I (NEB), to digest the DNA templates, followed by incubation at 37°C for 15 min. The reactions were centrifuged at 17,000 RPM for 10 min to pellet the precipitated pyrophosphate. The supernatant was loaded onto a Superose-6 Increase 10/300GL size exclusion column (GE Healthcare/Cytiva) equilibrated with 40 mM Hepes (pH 7.5), 50 mM KCl, and 5 mM MgCl_2_. The concentration of the cotranscriptionally folded RNA was determined by absorbance measurements at 260 nm on a DeNovix DS-11. The RNA keys were chemically synthesized and ordered from IDT (table S2).

### Fluorescence measurements

Measurements were performed in 384-well plates (Costar) with sample volumes of 50 μl containing 100 nM RNA, 500 nM DFHBI-1T, 40 mM Hepes, 50 mM KCl, and 5 mM MgCl_2_. DFHBI-1T was purchased from Lucerna Technologies. Fluorescence was monitored using a CLARIOstar Plus multi-mode microplate reader (BMG LABTECH) set at a constant temperature of 25°C. For the addition of RNA keys, the plates were removed from the plate reader, individual keys were added and mixed in the laboratory, and then the plates were reinserted into the machine for continued measurements. Excitation of DFHBI-1T was performed at 470 ± 10 nm and emission was recorded at 505 ± 10 nm for the time point measurements. For the excitation spectral scans, emission was recorded at 505 nm while excited from 420 to 480 nm. For the emission spectral scans, 470 nm was used for excitation and emission was recorded from 500 to 550 nm.

### Cryo-EM sample preparation and data collection

Samples were up-concentrated to ~2 mg/ml using Amicon Ultra centrifugal filters with a 30-kDa cutoff, spinning the samples at 14,000*g* at room temperature. ProtoChips Au-FLAT 1.2/1.3 300 mesh grids were glow-discharged for 45 s at 15 mA in a Pelco easiGlow before sample application. Grids were plunge-frozen using a Leica GP2, and the sample application chamber was kept at 100% humidity and 21°C. Three microliters of sample was applied to the grid which was then blotted with a manually calibrated stopping distance onto a double layer of Whatman #1 filter paper using a 4-s delay after application, 6 s of blot time, and 0 s of delay after blotting before plunging into liquid ethane at −184°C. Data were acquired at 300 keV on a Titan Krios G3i (Thermo Fisher Scientific) equipped with a K3 camera (Gatan/Ametek) and energy filter operated in EFTEM mode using a slit width of 20 eV. Data were collected over a defocus range of −0.5 to −2 μm with a targeted dose of 60 e^−^/Å^2^. Automated data collection was performed with EPU, and the data saved as gain normalized compressed .tiff files (K3) with a pixel size of 0.645 Å/pixel.

### Cryo-EM single-particle analysis data processing

CS-live was used to apply motion correction, CTF fitting, initial particle picking, and an initial ab initio model (*66*). The 3D volume from CS-live was used for a homogeneous refinement in CryoSPARC V3.2. Fifty 2D templates were created from this refined volume and templated particle picking was performed using these 50 templates. Particles were then extracted at a pixel size of ~2.7 Å/pixel and three ab initio models were generated using a subset of 30,000 randomly selected particles. A heterogeneous refinement using the three ab initio models and all the extracted particles was then performed. At this point, we had one junk class and two classes resembling our RNA origami trap. Another round of heterogeneous refinement was performed using the particles from the two good classes and three copies of the best 3D volume as initial search volumes. Approximately 550,000 particles from the two best classes were then used to start a five-class ab initio model search, followed by heterogeneous refinement into these five classes. This resulted in a subsection of 220,000 particles that were reaching the Nyquist limit set by our pixel size, 5.45 Å.

### Model building

Model building was performed in ChimeraX (*67*, *68*). The different motifs composing the 14-14 Traptamer were manually incorporated to fit into the cryo-EM volume. The crystal structure of iSpinach [Protein Data Bank (PDB): 5OB3] was truncated and fit. The crossovers, helical components, and tetraloops were generated using RNAbuild (*23*), leaving free 5′ and 3′ ends at the crossover junctions, and then manually positioned into the cryo-EM volume. The bKLs were generated by a combination of the NMR structure of the HIV-1 kissing loops (PDB: 2D1B) and a stem-loop generated with RNAbuild. Once helical placement was approximately correct, the individual components were joined using the “make bond” command from the ISOLDE (*69*) add-on to ChimeraX. The resulting PDB was renumbered using the PDB-Tools pdb-reres program (*70*) and then the correctly numbered PDB was sequence-corrected in ChimeraX using the swapNA command. This model was then passed through real-space refinement (RSR) in Phenix (*71*–*73*) (using default parameters, our best-refined volume, and the resolution supplied by the FSC curve at a 0.143 cutoff) to remove any severe clashes that ISOLDE could not handle. The models were then inspected in ChimeraX and subjected to further refinement using molecular dynamics flexible fitting with Visual Molecular Dynamics using ISOLDE. A final round of RSR in Phenix was performed to optimize the backbone angles. Validation of the goodness of fit between model and map was performed using the Phenix validation tool (*73*).

### Statistical analysis

Statistical analysis of the fluorescence measurements was done in the GraphPad Prism software. Experiments were done as three technical replicates to obtain error bars that are represented as the means ± SD.
