# Supplementary material for: An RNA origami robot that traps and releases a fluorescent aptamer
Source: Sci Adv. 2024 Mar 20;10(12):eadk1250. doi: 10.1126/sciadv.adk1250 (PMC10954211; doi:10.1126/sciadv.adk1250)
Supplement: Supplementary file 1 — Figs. S1 to S12 Tables S1 and S2 [file sciadv.adk1250_sm.pdf]

Science Advances
AAAS

Supplementary Materials for

An RNA origami robot that traps and releases a fluorescent aptamer

Néstor Sampedro Vallina *et al.*

Corresponding author: Ebbe S. Andersen, esa@inano.au.dk

Sci. Adv. **10**, eadk1250 (2024)
DOI: 10.1126/sciadv.adk1250

This PDF file includes:

Figs. S1 to S12
Tables S1 and S2

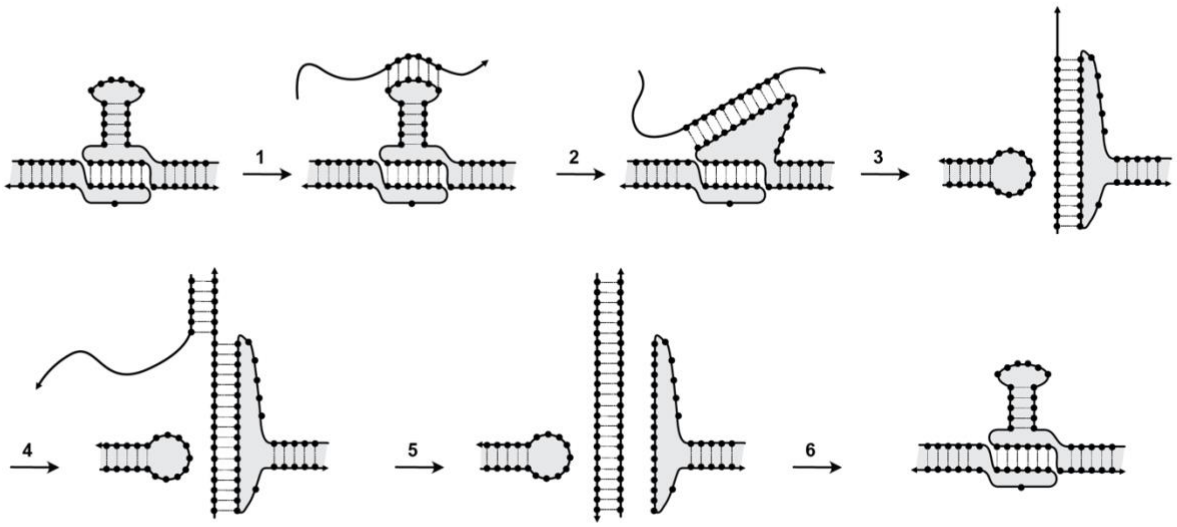

Fig. S1. Proposed mechanism for loop-mediated strand displacement in a bKL.

A bKL-KL interaction is shown where the branch forms a hairpin with an apical loop. In step 1, an RNA key strand anneals to the apical loop. In step 2, the RNA key strand invades into the stem of the hairpin. In step 3, the RNA key strand further invades into the KL thus breaking the bKL-KL interaction. In step 4, an RNA anti-key strand is added that anneals to the 3' toehold of the RNA key strand. In step 5, the RNA anti-key strand invades the RNA key strand bKL stem to release a fully hybridized waste product. In step 6, the bKL-KL interaction reforms.

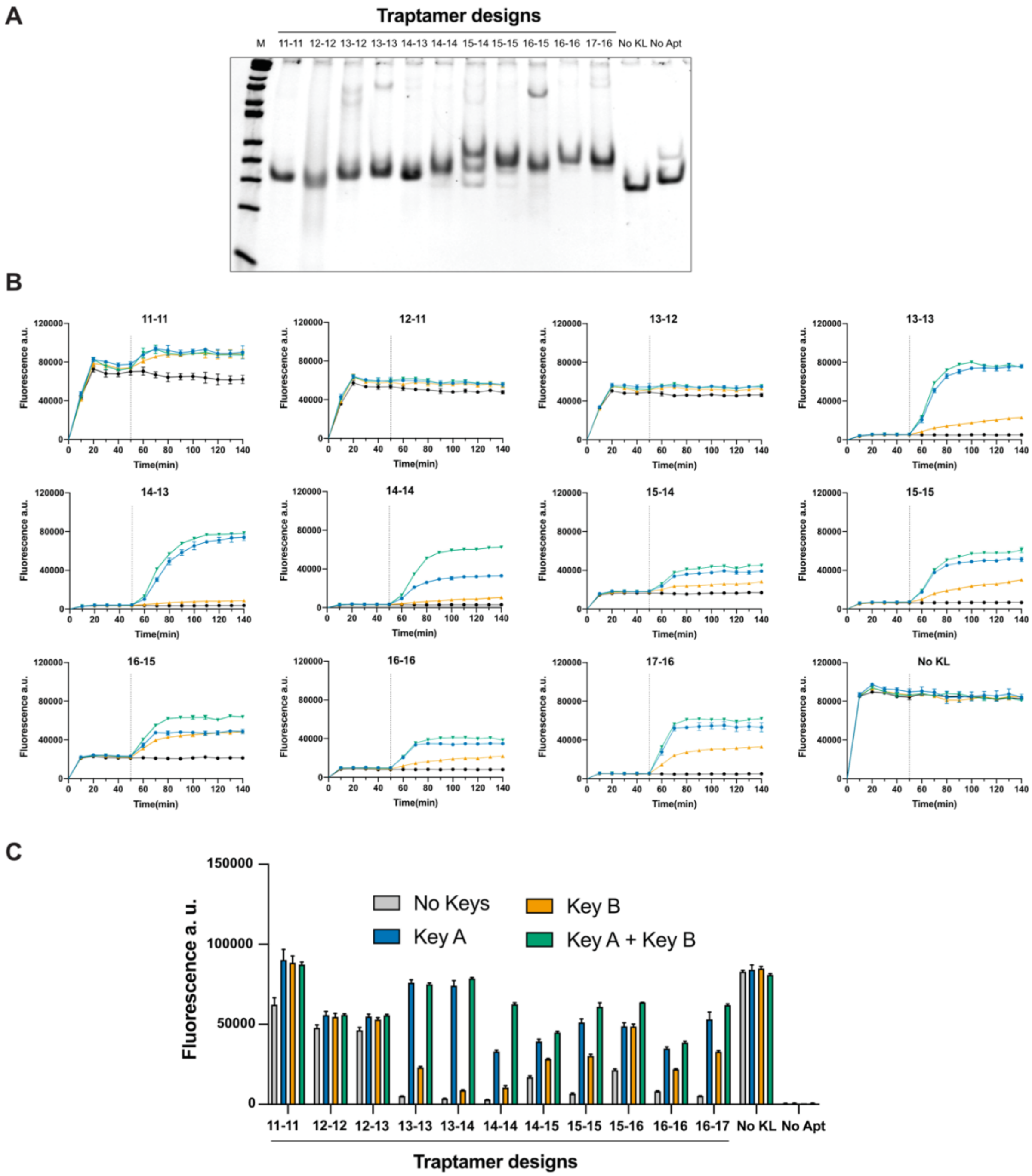

Fig. S2. Design screening for a co-transcriptionally folded RNA mechanical trap.

(**A**) 8% native PAGE gel showing the mobility profile of Traptamer designs after SEC purification. M indicates marker lane with 1kb plus DNA ladder from NEB. (**B**) Monitored fluorescence over time of the different designs at 100 nM RNA concentration at 25 °C. The fluorophore DFHBI-1T (500 nM) was added after the first measurement and 5X (500 nM) single stranded RNA keys were added after 50 minutes (black dashed line). Data corresponds to 3 technical replicates; error bars represent mean ± SD. (**C**) Fluorescence observed at 140 mins (85 minutes upon addition of the RNA keys) for the different designs.

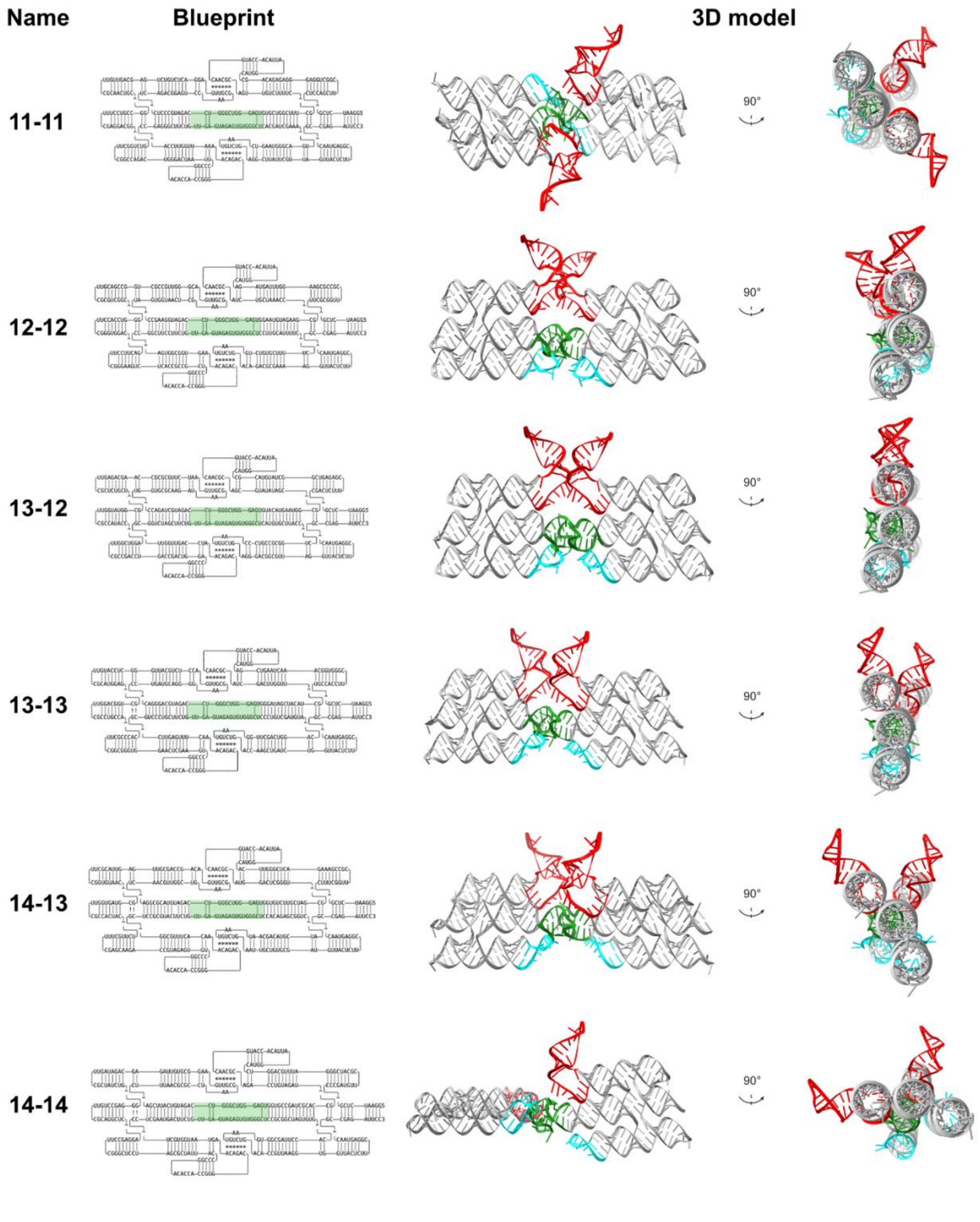

Fig. S3. Blueprints and 3D models of the Traptamer designs (continues next page).
Blueprints are shown with the iSpinach motif marked in green. 3D models are shown in two perpendicular views with bKL in red, KL in cyan, and iSpinach in green.

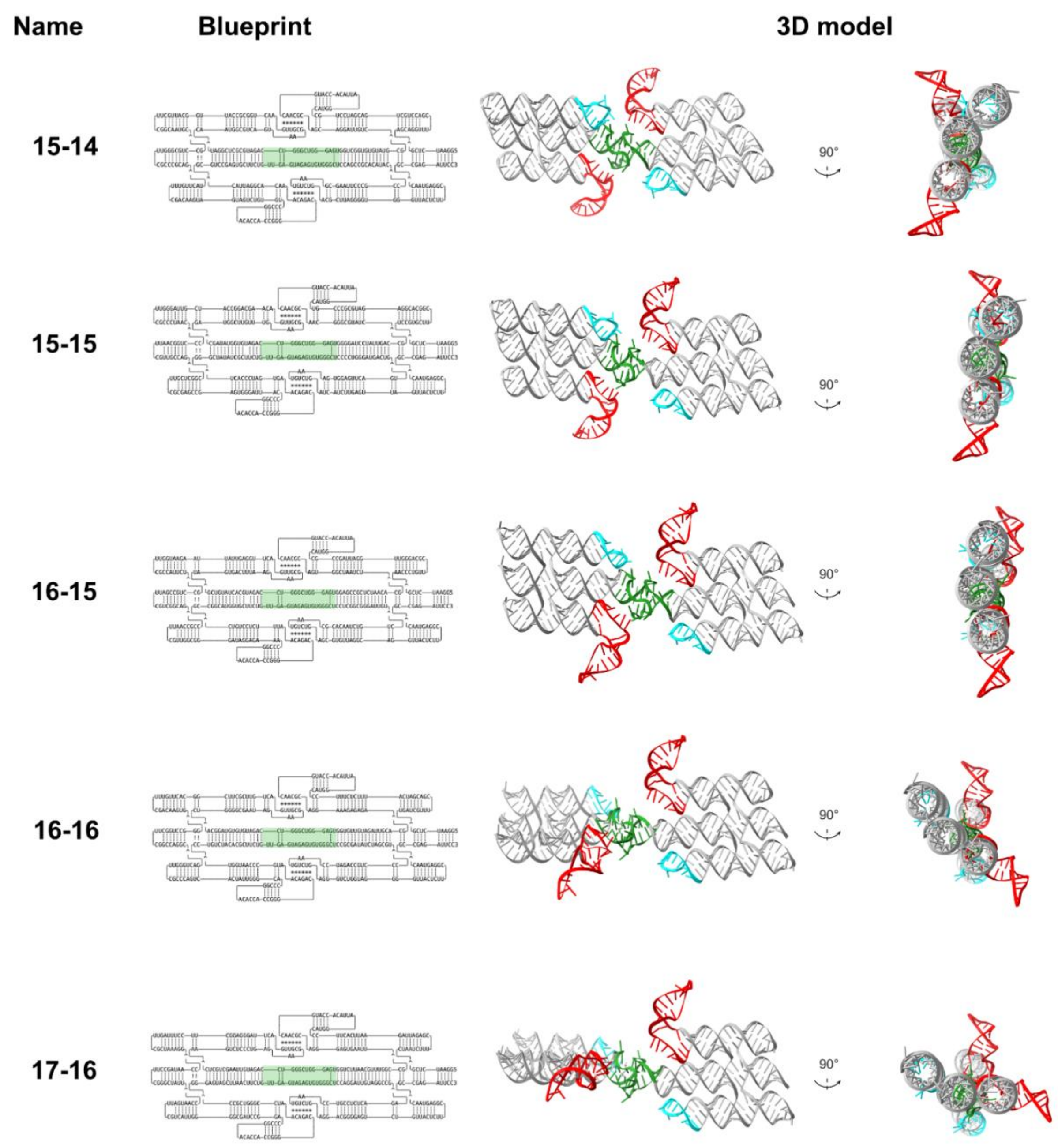

Fig. S3. (Continued).

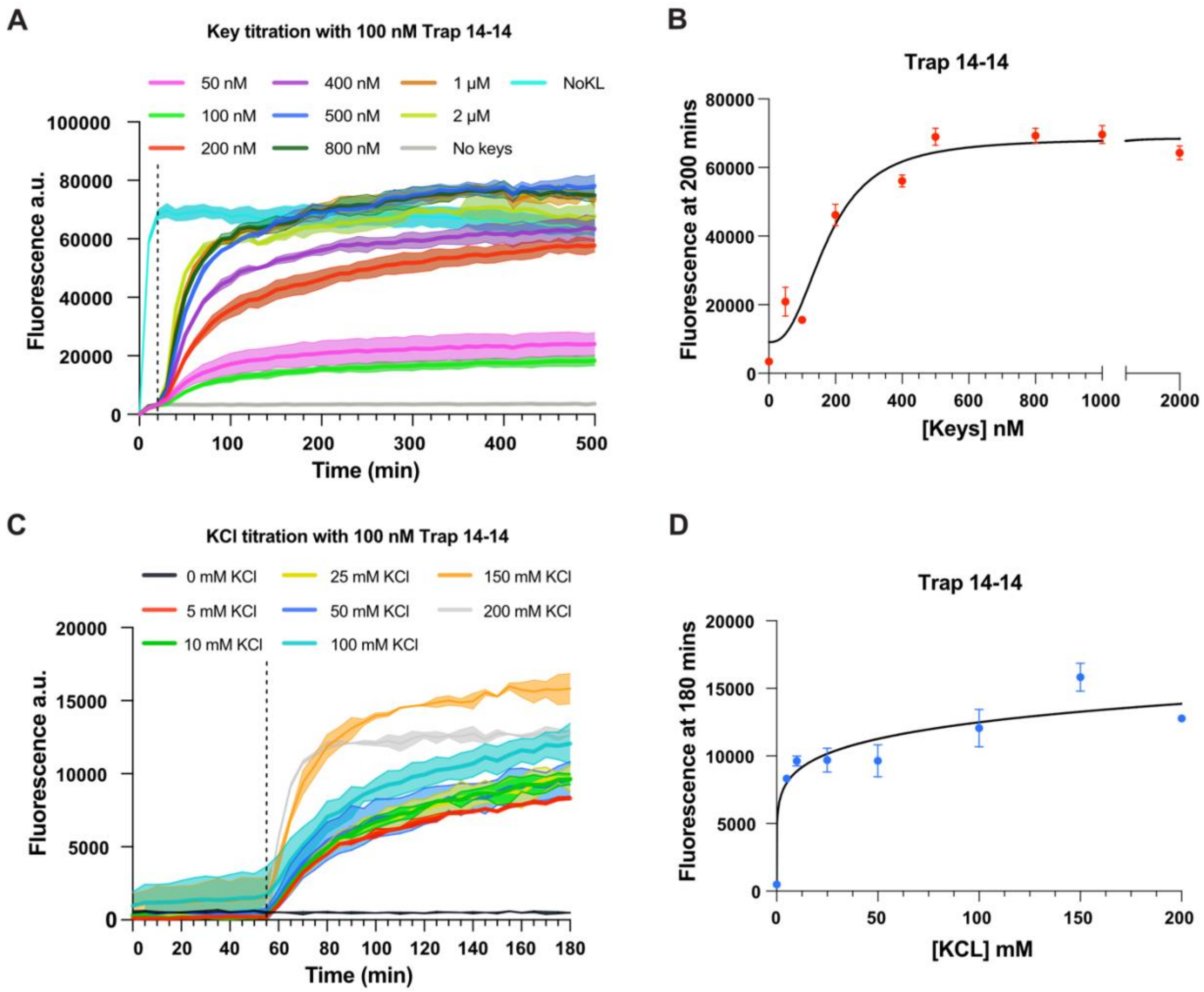

Fig. S4. Responses to key and KCl concentration of the Traptamer 14-14 design.

(**A**) ssRNA key titration. Monitored fluorescence over time of Traptamer 14-14 RNA (100 nM) in complex with DFHBI-1T (500 nM) at 25 °C. Different concentrations of ssRNAs were added after the first fluorescence measurement. The No kissing loops (NoKL) positive control is shown in cyan blue. (**B**) Fluorescence measured after 200 minutes upon addition of different concentrations of ssRNA keys from panel A. (**C**) KCl titration. Monitored fluorescence over time of Traptamer 14-14 (100 nM) in buffers with different KCl concentrations 25 °C. (**D**) Fluorescence after 180 minutes upon addition of keys at different KCl concentrations from panel C. All data shown corresponds to 3 technical replicates and error bars represent mean ± SD.

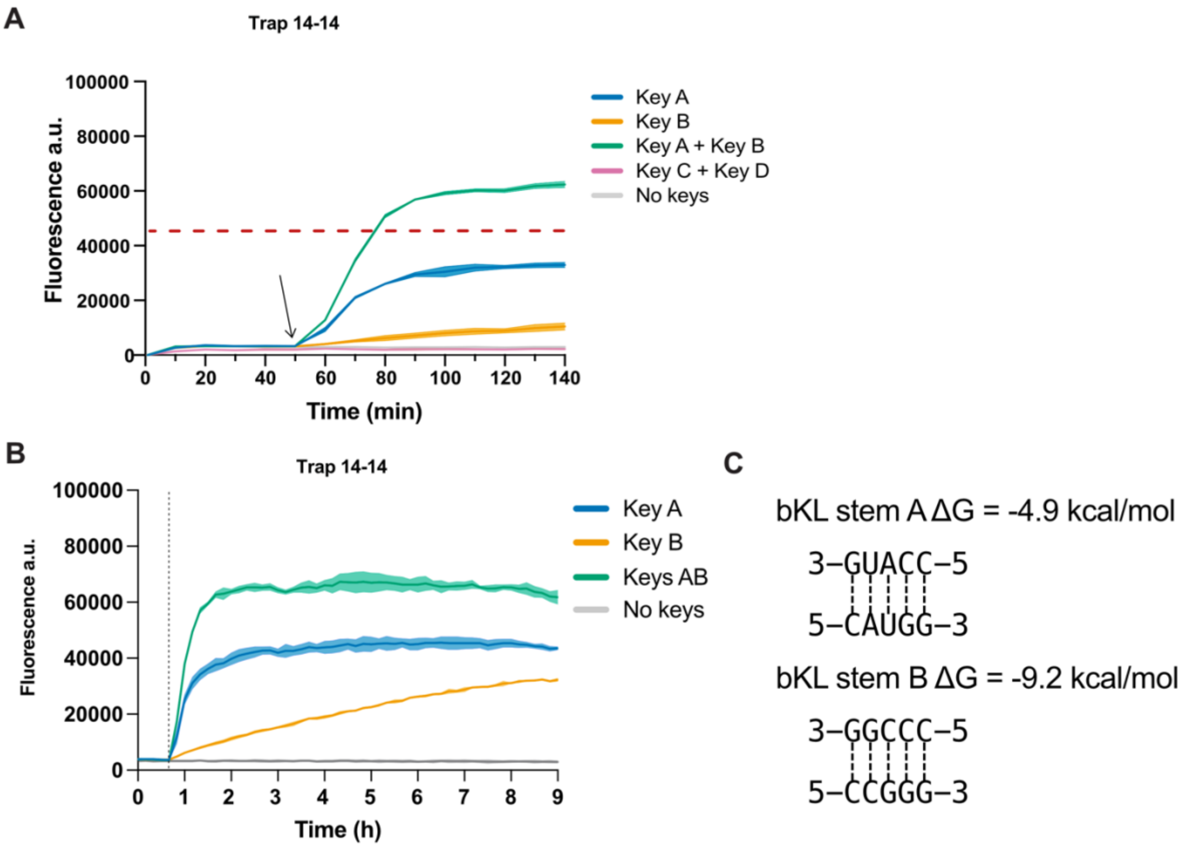

Fig. S5. Opening of Traptamer 14-14 and stabilities over extended time period.
(**A**) Observed fluorescence for the 14-14 device over time. DFHBI-1T (500 nM) was added after the first measurement, cognate and negative control keys (500 nM) were added after 45 minutes (dashed line) at 25 °C. Data corresponds to 3 technical replicates; error bars represent mean ± SD. (**B**) Monitored fluorescence over 9 hours of Traptamer 14-14 (100 nM) in solution with DFHBI-1T (500 nM) after addition of RNA keys (500 nM) at 25 °C (dashed line indicates key addition). Data represents 3 technical replicates ± SD. (**C**) Free energies of the bKL stems calculated with NUPACK.

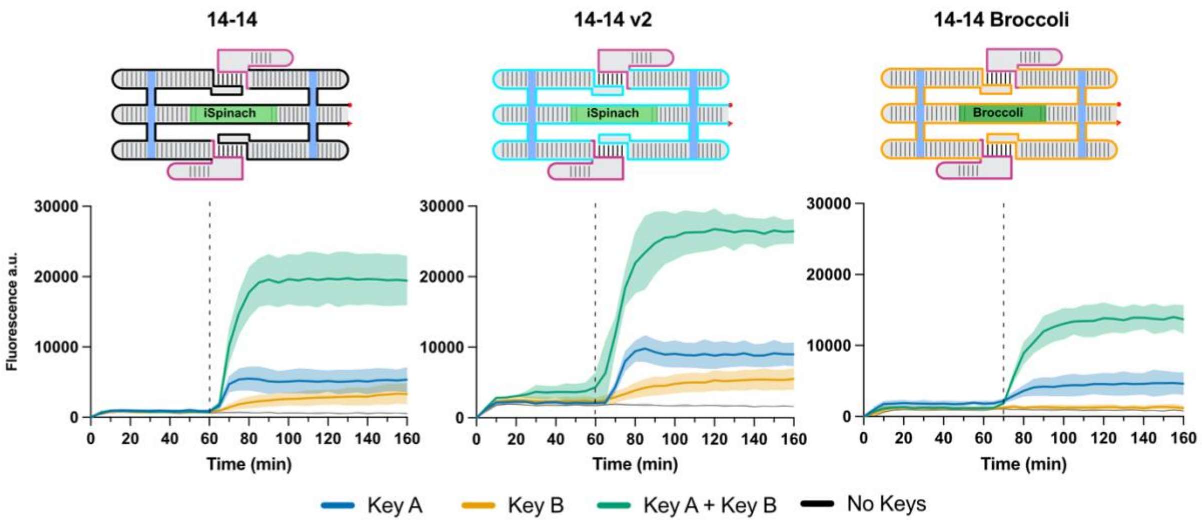

Fig. S6. Function of Traptamer 14-14 with different sequence designs and Broccoli aptamer.

Traptamer 14-14 and 14-14 v2 have different scaffold sequences (black vs cyan line). Traptamer 14-14 Broccoli has both the Broccoli aptamer and a different scaffold sequence (orange line). Monitored fluorescence with 100 nM RNA, 500 nM DFHBI-1T, and 500 nM RNA keys (500 nm) at 25 °C (dashed line indicates key addition). Key A (blue), Key B (orange), Key A + Key B (green). Data represents 3 technical replicates ± SD.

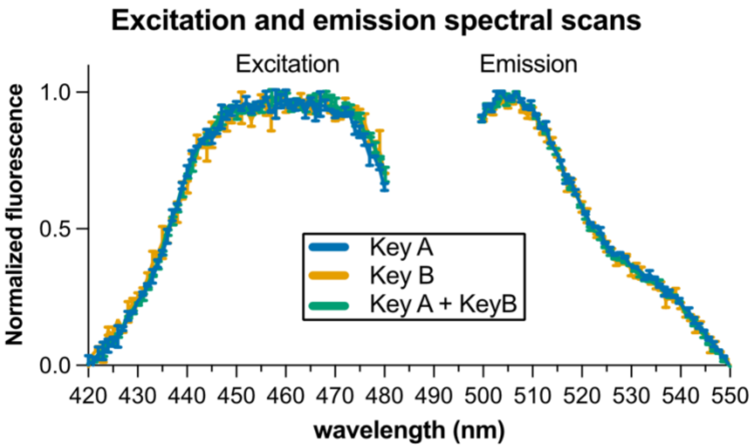

Fig. S7. No effect of key addition on excitation and emission spectra.
Excitation and emission spectra of DFHBI-1T (500 nM) in complex with the Traptamer 14-14 (100 nM) at different key conditions (500 nM) at 25 °C.

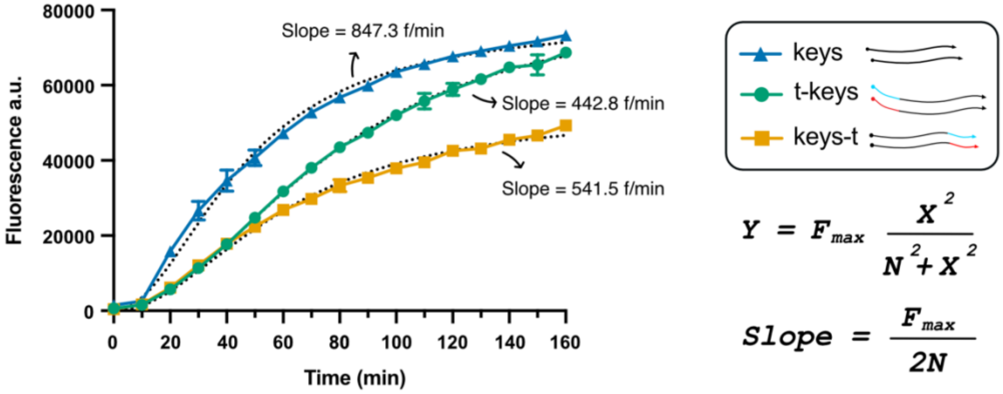

$$Y = F_{max} \frac{X^2}{N^2 + X^2}$$

$$Slope = \frac{F_{max}}{2N}$$

Fig. S8. Effect of adding toeholds on 3'-end of RNA keys.

Fluorescence activation of 100 nM Traptamer 14-14 after addition of keys (500 nM) with no toehold or toehold placed at 5'- (t-keys) or 3'- (keys-t) ends (500 nM) at 25 °C. Data represents 3 technical replicates ± SD. *Fmax* represents the maximum fluorescence and *N* represents the inflection point, i.e., time point (x) at which fluorescence has its maximum increase. Model fit R^2 = 0.993 for keys, R^2 = 0.998 for t-keys and R^2 = 0.993 for keys-t.

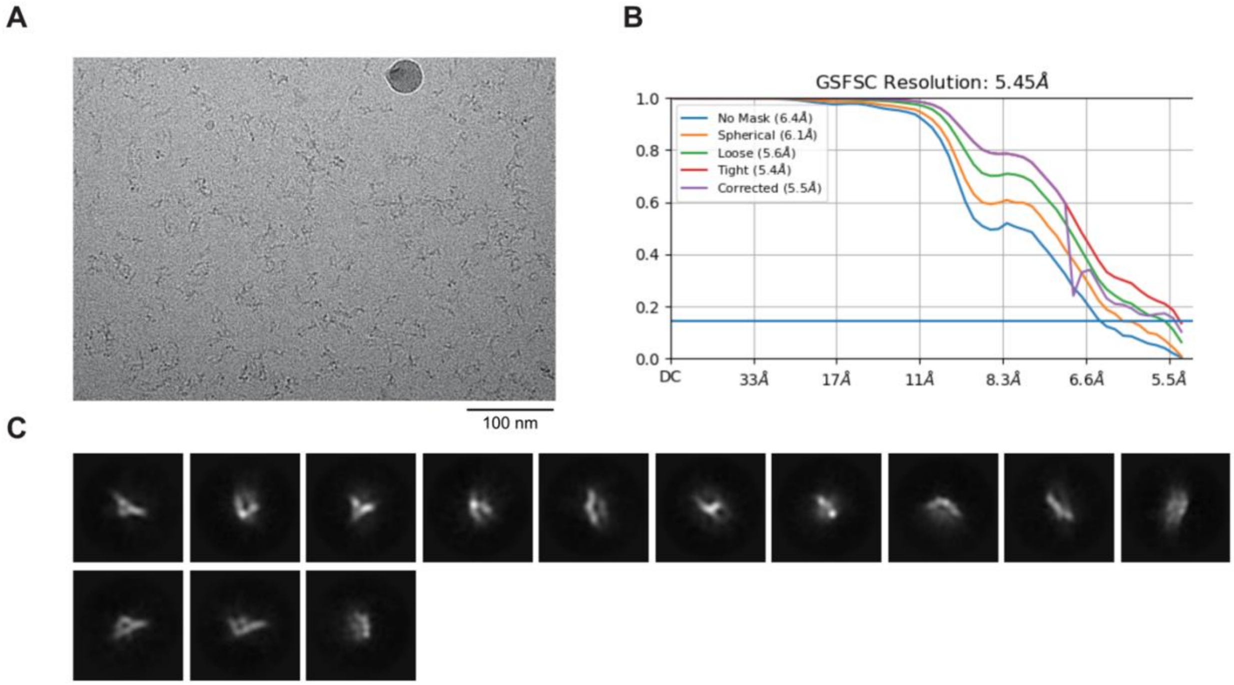

Fig. S9. Cryo-EM analysis of Traptamer 14-14 with no keys.

(**A**) Example cryo-EM micrograph from the locked Traptamer 14-14 dataset. (**B**) Gold-standard Fourier Shell Correlation for the locked state of Traptamer 14-14. (**C**) 2D Class. Averages from the final particle stack of the locked Traptamer 14-14 dataset. Box size: 50 x 50 nm.

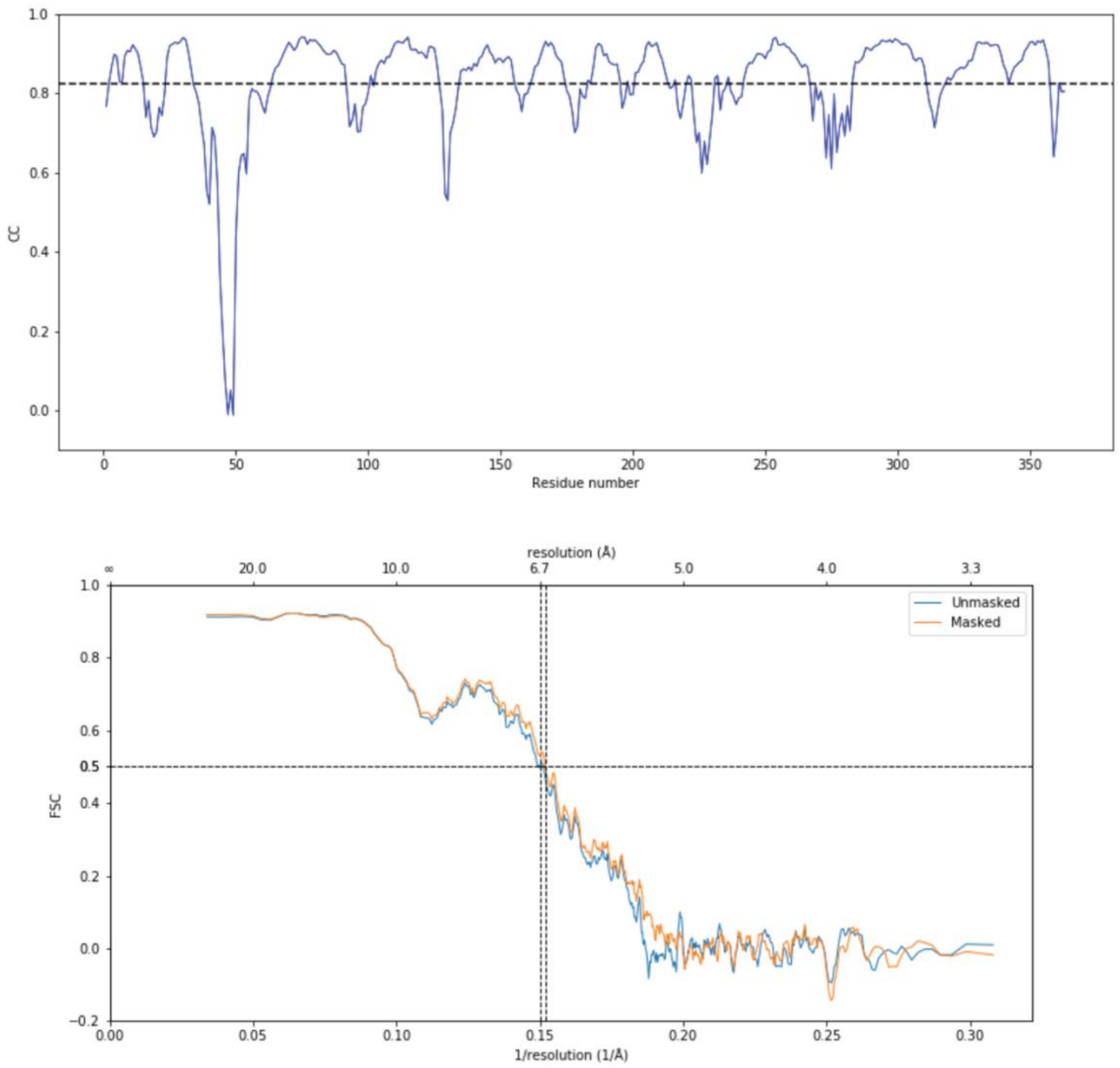

Fig. S10. Cross Correlation and Fourier Shell Correlation.

Per residue Cross Correlation (top) and Fourier Shell Correlation (bottom) between atomic model and cryo-EM map for the Traptamer 14-14 with no keys after Phenix RSR.

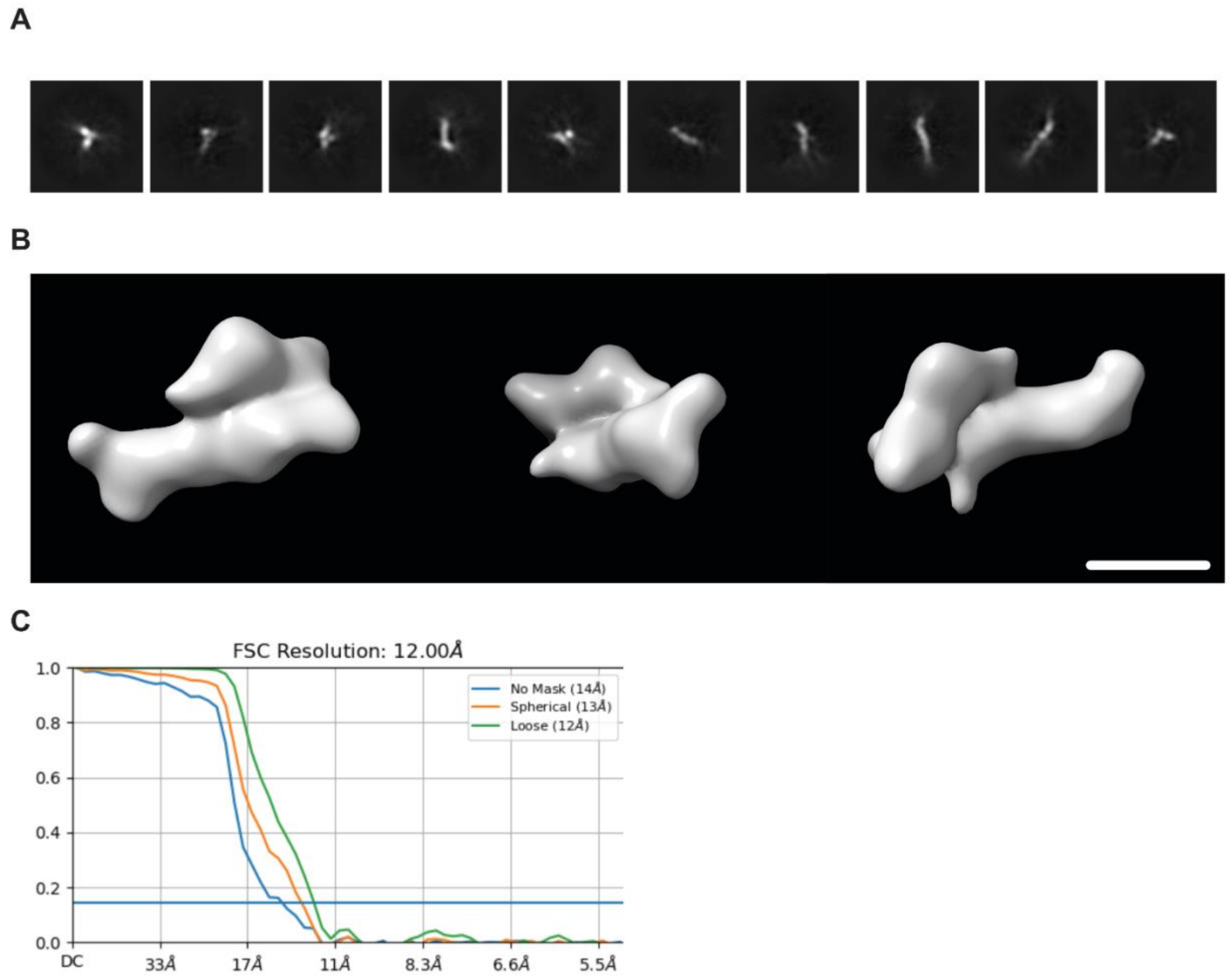

Fig. S11. Cryo-EM analysis of Traptamer 14-14 upon addition of keys.

(**A**) 2D Class averages from the. Final particle stack of the open Traptamer 14-14. dataset. Box size: 50 x 50 nm. (**B**) Three views of the Trap-14-14 device with keys reconstruction. Scale bar: 10 nm. (**C**) Gold-Standard Fourier Shell Correlation for the open state of Traptamer 14-14.

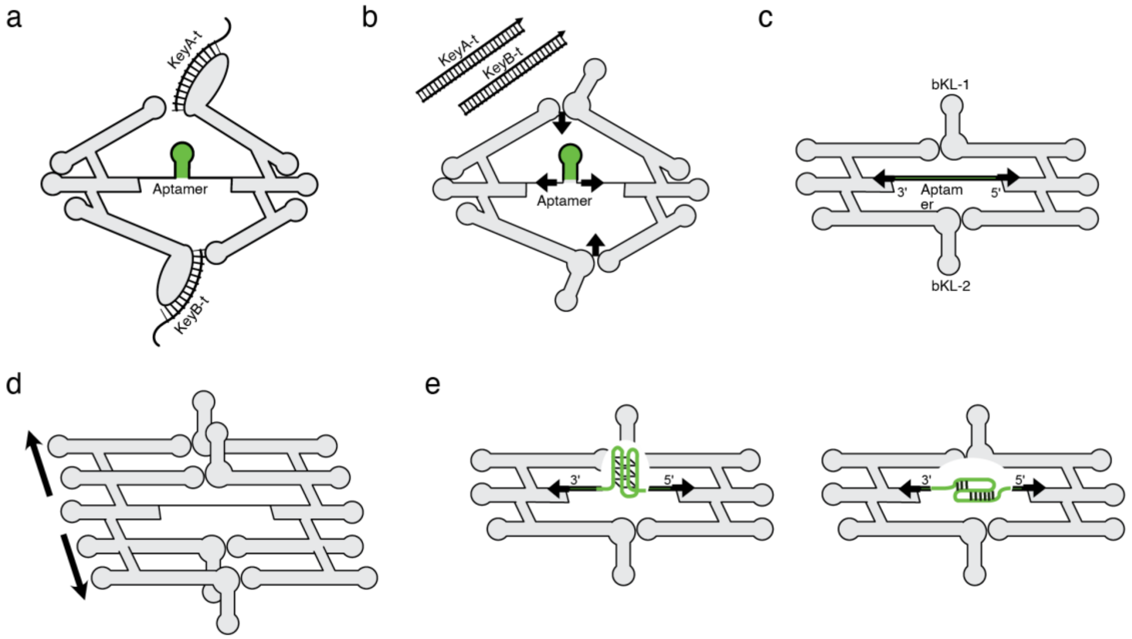

Fig. S12. Suggestion for Traptamer pulling configuration.

(**A**) Traptamer in the open state with a single stranded central connection that folds into an aptamer. (**B**) When adding displacement keys, the bKL-KL interactions can start forming inducing a force on the central aptamer. (**C**) When the bKL-KL interactions are fully formed they exert a strong force on the central strand causing the aptamer to unfold. (**D**) Illustration of an extended Traptamer, where more bKL-KL interactions work together to induce a stronger force on the central single strand. (**E**) Examples of other RNA motifs that can be installed in the pulling device: a G-quadruplex and an H-type pseudoknot.

Table S1. RNA blueprints and sequences.

No Kissing loops control (NoKL)

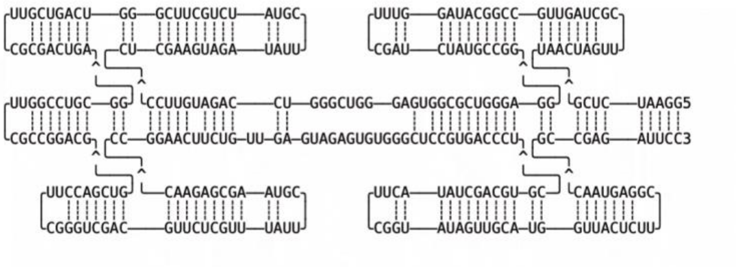

GGAAUCUCGUAACUAGUUCGCUAGUUGCCGGCAUAGGUUUCGAUCUAUGCCGGGCAGGGUCGCGGUGAGGGUCGGGUCCAGAUGUUCCCUCGAAGUAGAUA
UUCGUAUCUGCUUCGGGUCAGUCGUUCGCGACUGAGGCGUCCGGUUCGCCGGACGGUCGACCUUCGGGUCGACGUUCUCGUUUAUUCGUAAGCGAGAACCC
GGAACUUCUGUUGAGUAGAGUGUGGGCUCCGUGACCCUCGUGCAGCUAUACUUCGGUAUAGUUGCAUGGUUACUCUUCGGAGUAACGCCGAGAUUCC

No Aptamer control (NoApt)

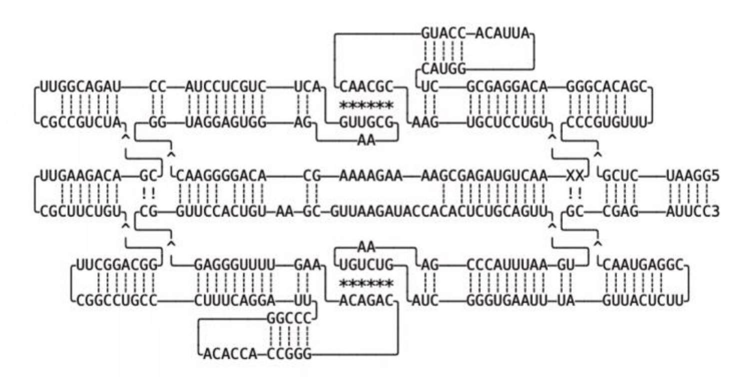

GGAAUCUCGCCCGUGUUUCGACACGGGACAGGAGCGCUCAUGGAUUACACCAUGCAACGCAAGUGCUCCUGUGCAACUGUAGAGCGAAAAGAAAAGCACAG
GGGAACGGUAGGAGUGGAGAAGCGUUGACUCUGCUCCUACCUAGACGGUUCGCCGUCUACGACAGAAGUUCGCUUCUGUGGCAGGCUUCGGCCUGCCCUUU
CAGGAUUCCCGGACACCACCGGGCAGACAAAGUUUUGGGAGCGGUUCCACUGUAAGCGUUAAGAUACCACACUCUGCAGUUUGAAUUUACCCGAAAUGUCU
GAUCGGGUGAAUUUAGUUACUCUUCGGAGUAACGCCGAGAUUCC

Trap-11-11

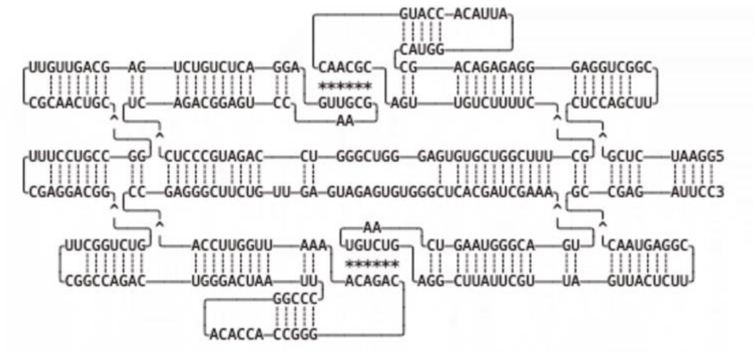

GGAAUCUCGCUCCAGCUUCGGCUGGAGGGAGAGACAGCCAUGGAUUACACCAUGCAACGCAGUUGUCUUUUCGCUUUCGGUCGUGUGAGGGUCGGGUCCAG
AUGCCCUCUCAGACGGAGUCCAAGCGUUGAGGACUCUGUCUGAGCAGUUGUUCGCAACUGCGGCCGUCCUUUCGAGGACGGGUCUGGCUUCGGCCAGACUG
GGACUAAUUCCCGGACACCACCGGGCAGACAAAAUUGGUUCCACCGAGGGCUUCUGUUGAGUAGAGUGUGGGCUCACGAUCGAAAUGACGGGUAAGUCAAU
GUCUGAGGCUUAUUCGUUAGUUACUCUUCGGAGUAACGCCGAGAUUCC

Trap-12-12

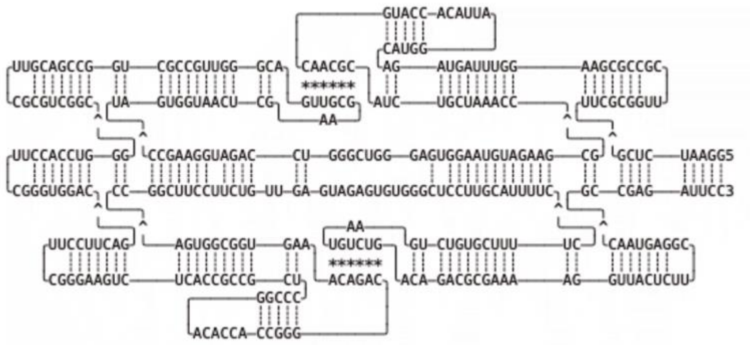

GGAAUCUCGUUCGCGGUUCGCCGCGAAGGUUUAGUAGACAUGGAUUACACCAUGCAACGCAUCUGCUAAACCGCGAAGAUGUAAGGUGAGGGUCGGGUCCA
GAUGGAAGCCUAGUGGUAACUCGAAGCGUUGACGGGUUGCCGCUGGCCGACGUUCGCGUCGGCGGGUCCACCUUCGGGUGGACGACUUCCUUCGGGAAGUC
UCACCGCCGCUCCCGGACACCACCGGGCAGACAAAGUGGCGGUGACCGGCUUCCUUCUGUUGAGUAGAGUGUGGGCUCCUUGCAUUUUCCUUUUCGUGUCU
GAAUGUCUGACAGACGCGAAAAGGUUACUCUUCGGAGUAACGCCGAGAUUCC

Trap-13-12

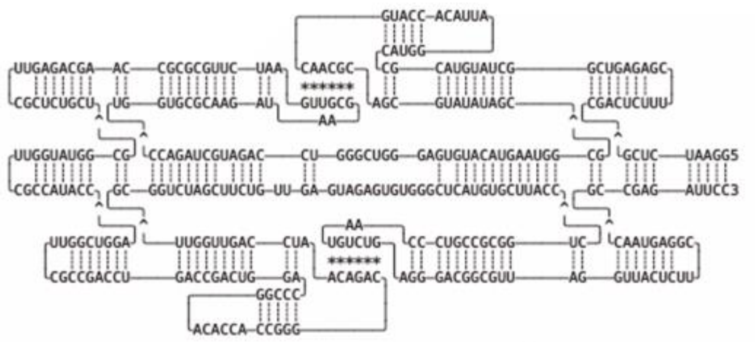

GGAAUCUCGCGACUCUUUCGAGAGUCGGCUAUGUACGCCAUGGAUUACACCAUGCAACGCAGCGUAUAUAGCGCGGUAAGUACAUGUGAGGGUCGGGUCCA
GAUGCUAGACCUGGUGCGCAAGAUAAGCGUUGAAUCUUGCGCGCCAAGCAGAGUUCGCUCUGCUGCGGUAUGGUUCGCCAUACCAGGUCGGUUCGCCGACC
UGACCGACUGGACCCGGACACCACCGGGCAGACAAUCCAGUUGGUUGCGGUCUAGCUUCUGUUGAGUAGAGUGUGGGCUCAUGUGCUUACCCUGGCGCCGU
CCCAAUGUCUGAGGGACGGCGUUAGGUUACUCUUCGGAGUAACGCCGAGAUUCC

Trap-13-13

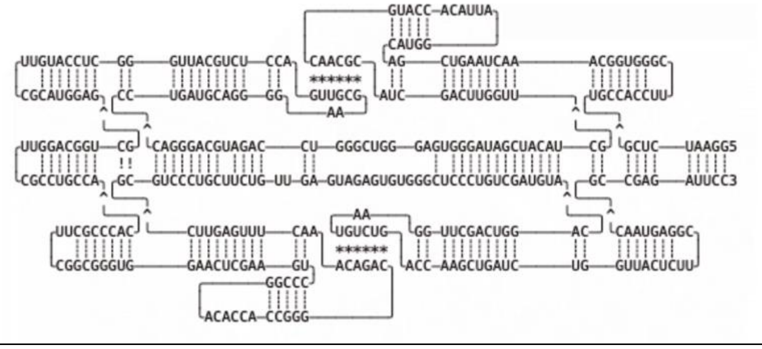

GGAAUCUCGUGCCACCUUCGGGUGGCAAACUAAGUCGACAUGGAUUACACCAUGCAACGCAUCGACUUGGUUGCUACAUCGAUAGGGUGAGGGUCGGGUCC
AGAUGCAGGGACCCUGAUGCAGGGGAAGCGUUGACCUCUGCAUUGGGCUCCAUGUUCGCAUGGAGGCUGGCAGGUUCGCCUGCCACACCCGCUUCGGCGGG
UGGAACUCGAAGUCCCGGACACCACCGGGCAGACAAACUUUGAGUUCGCGUCCCUGCUUCUGUUGAGUAGAGUGUGGGCUCCCUGUCGAUGUACAGGUCAG
CUUGGAAUGUCUGACCAAGCUGAUCUGGUUACUCUUCGGAGUAACGCCGAGAUUCC

Trap-14-13
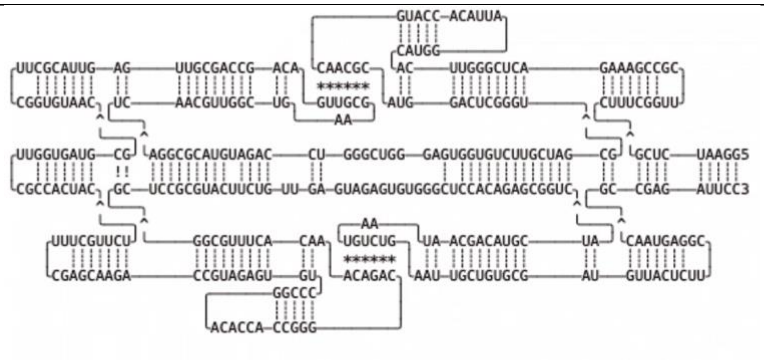
GGAAUCUCGCUUUCGGUUCGCCGAAAGACUCGGGUUCACAUGGAUUACACCAUGCAACGCAUGGACUCGGGUGCGAUCGUUCUGUGGUGAGGGUCGGGUCC AGAUGUACGCGGAUCAACGUUGGCUGAAGCGUUGACAGCCAGCGUUGAGUUACGCUUCGGGUGUAACGCGUAGUGGUUCGCCACUACUCUUGCUUUCGAGCA AGACCGUAGAGUGUCCCGGACACCACCGGGCAGACAAACACUUUGCGGGCUCCGCGUACUUCUGUUGAGUAGAGUGUGGGCUCCACAGAGCGGUCAUCGUA CAGCAAUAAUGUCUGAAUUGCUGUGCGAUGUUACUCUUCGGAGUAACGCCGAGAUUCC

Trap-14-14
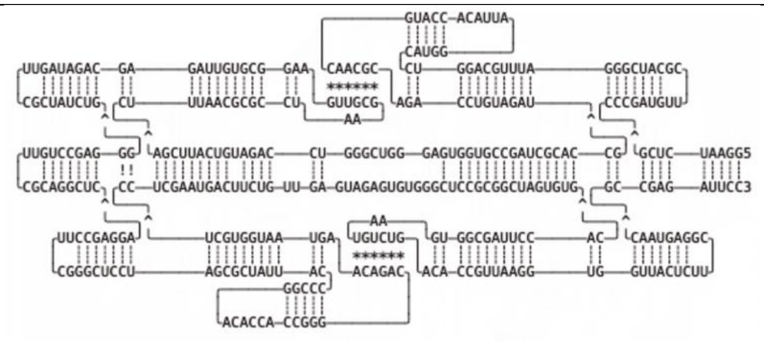
GGAAUCUCGCCCGAUGUUCGCAUCGGGAUUUGCAGGUCCAUGGAUUACACCAUGCAACGCAGACCUGUAGAUGCCACGCUAGCCGUGGUGAGGGUCGGGUC CAGAUGUCAUUCGACUUUAACGCGCCUAAGCGUUGAAGGCGUGUUAGAGCAGAUAGUUCGCUAUCUGGGGAGCCUGUUCGCAGGCUCAGGAGCCUUCGGGC UCCUAGCGCUAUUACCCCGGACACCACCGGGCAGACAAGUAAUGGUGCUCCUCGAAUGACUUCUGUUGAGUAGAGUGUGGGCUCCGCGGCUAGUGUGCACC UUAGCGGUGAAUGUCUGACACCGUUAAGGUGGUUACUCUUCGGAGUAACGCCGAGAUUCC

Trap-15-14
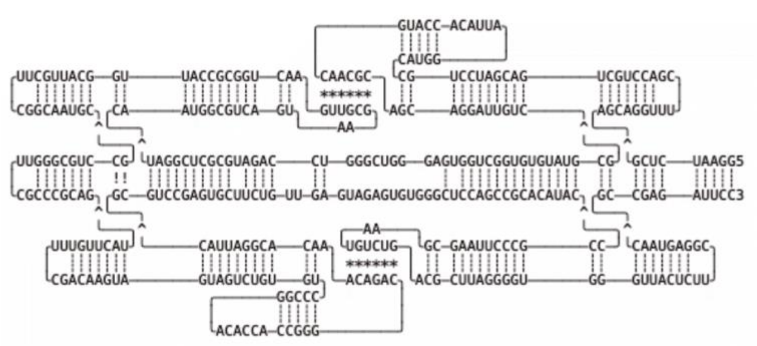
GGAAUCUCGAGCAGGUUUCGACCUGCUGACGAUCCUGCCAUGGAUUACACCAUGCAACGCAGCAGGAUUGUCGCGUAUGUGUGGCUGGUGAGGGUCGGGUC CAGAUGCGCUCGGAUCAAUGGCGUCAGUAAGCGUUGAACUGGCGCCAUUGGCAUUGCUUCGGCAAUGCGCCUGCGGGUUCGCCCGCAGUACUUGUUUCGAC AAGUAGUAGUCUGUGUCCCGGACACCACCGGGCAGACAAACACGGAUUACGCGUCCGAGUGCUUCUGUUGAGUAGAGUGUGGGCUCCAGCCGCACAUACCC GCCCUUAAGCGAAUGUCUGACGCUUAGGGGUGGGUUACUCUUCGGAGUAACGCCGAGAUUCC

Trap-15-15

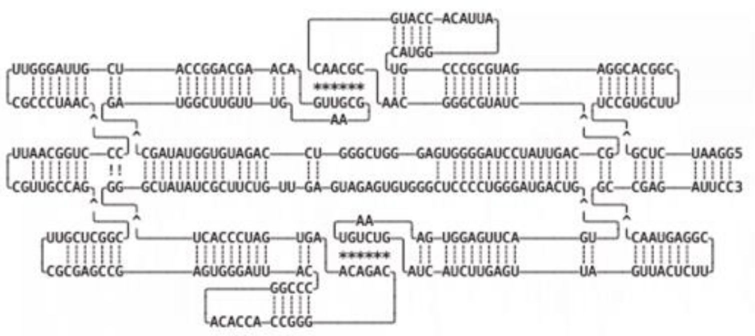

GGAAUCUCGUCCGUGCUUCGGCACGGAGAUGCGCCCGUCAUGGAUUACACCAUGCAACGCAACGGGCGUAUCGCCAGUUAUCCUAGGGGUGAGGGUCGGGU
CCAGAUGUGGUAUAGCGAUGGCUUGUUUGAAGCGUUGACAAGCAGGCCAUCGUUAGGGUUCGCCCUAACCCCUGGCAAUUCGUUGCCAGCGGCUCGUUCGC
GAGCCGAGUGGGAUUACCCCGGACACCACCGGGCAGACAAGUGAUCCCACUGGGCUAUAUCGCUUCUGUUGAGUAGAGUGUGGGCUCCCCUGGGAUGACUG
UGACUUGAGGUGAAAUGUCUGAUCAUCUUGAGUUAGUUACUCUUCGGAGUAACGCCGAGAUUCC

Trap-16-15

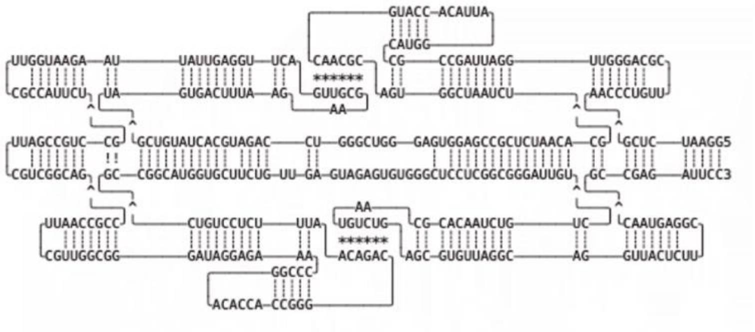

GGAAUCUCGAACCCUGUUCGCAGGGUUGGAUUAGCCGCCAUGGAUUACACCAUGCAACGCAGUGGCUAAUCUGCACAAUCUCGCCGAGGUGAGGGUCGGGU
CCAGAUGCACUAUGUCGUAGUGACUUUAAGAAGCGUUGACUUGGAGUUAUUAAGAAUGGUUCGCCAUUCUGCCUGCCGAUUCGUCGGCAGCCGCCAAUUCG
UUGGCGGGAUAGGAGAAACCCGGACACCACCGGGCAGACAAUUUCUCCUGUCGCCGGCAUGGUGCUUCUGUUGAGUAGAGUGUGGGCUCCUCGGCGGGAUU
GUCUGUCUAACACGCAAUGUCUGAGCGUGUUAGGCAGGUUACUCUUCGGAGUAACGCCGAGAUUCC

Trap-16-16

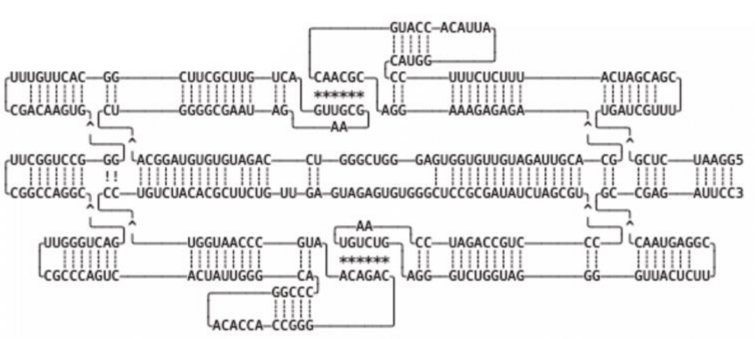

GGAAUCUCGUGAUCGUUUCGACGAUCAUUUCUCUUUCCCAUGGAUUACACCAUGCAACGCAGGAAAGAGAGAGCACGUUAGAUGUUGUGGUGAGGGUCGGG
UCCAGAUGUGUGUAGGCACUGGGGCGAAUAGAAGCGUUGACUGUUCGCUUCGGCACUUGUUUCGACAAGUGGGGCCUGGCUUCGGCCAGGCGACUGGGUUC
GCCCAGUCACUAUUGGGCACCCGGACACCACCGGGCAGACAAUGCCCAAUGGUCCUGUCUACACGCUUCUGUUGAGUAGAGUGUGGGCUCCGCGAUAUCUA
GCGUCCCUGCCAGAUCCAAUGUCUGAGGGUCUGGUAGGGGUUACUCUUCGGAGUAACGCCGAGAUUCC

Trap-17-16

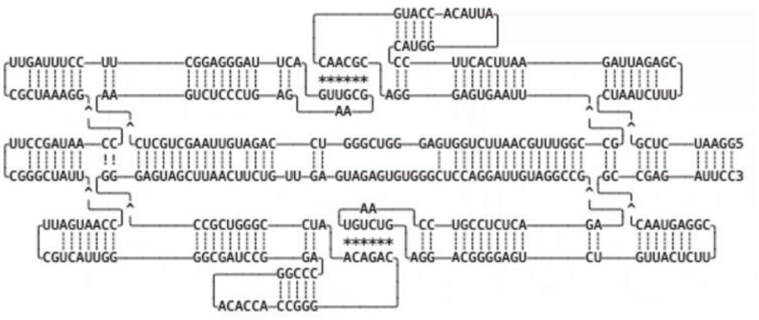

GGAAUCUCGCUAAUCUUUCGAGAUUAGAAUUCACUUCCCAUGGAUUACACCAUGCAACGCAGGGAGUGAAUUGCCGGUUUGCAAUUCUGGUGAGGGUCGGG
UCCAGAUGUUAAGCUGCUCAAGUCUCCCUGAGAAGCGUUGACUUAGGGAGGCUUCCUUUAGUUCGCUAAAGGCCAAUAGCCUUCGGGCUAUUCCAAUGAUU
CGUCAUUGGGGCGAUCCGGACCCGGACACCACCGGGCAGACAAUCCGGGUCGCCGGGAGUAGCUUAACUUCUGUUGAGUAGAGUGUGGGCUCCAGGAUUGU
AGGCCGAGACUCUCCGUCCAAUGUCUGAGGACGGGGAGUCUGUUACUCUUCGGAGUAACGCCGAGAUUCC

Trap-14-14 v2

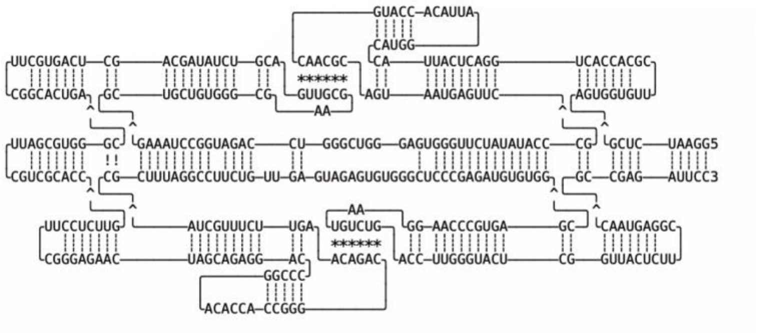

GGAAUCUCGAGUGGUGUUCGCACCACUGGACUCAUUACCAUGGAUUACACCAUGCAACGCAGUAAUGAGUUCGCCCAUAUAUCUUGGGUGAGGGUCGGGUC
CAGAUGGCCUAAAGGCUGCUGUGGGCGAAGCGUUGACGUCUAUAGCAGCUCAGUGCUUCGGCACUGACGGGUGCGAUUCGUCGCACCGUUCUCCUUCGGGA
GAACUAGCAGAGGACCCCGGACACCACCGGGCAGACAAGUUCUUUGCUACGCUUUAGGCCUUCUGUUGAGUAGAGUGUGGGCUCCCGAGAUGUGUGGCGAG
UGCCCAAGGAAUGUCUGACCUUGGGUACUCGGUUACUCUUCGGAGUAACGCCGAGAUUCC

Trap-14-14 Broccoli

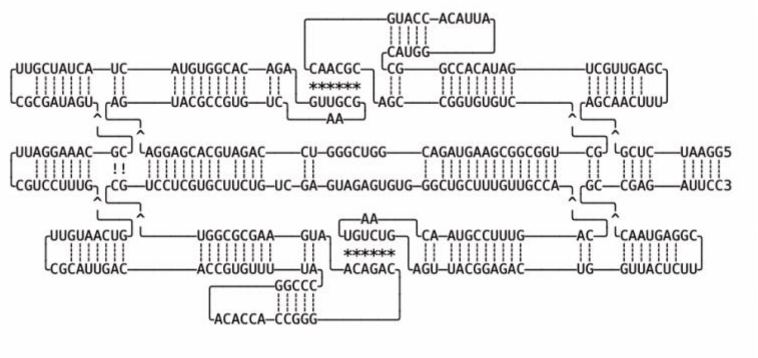

GGAAUCUCGAGCAACUUUCGAGUUGCUGAUACACCGGCCAUGGAUUACACCAUGCAACGCAGCCGGUGUGUCGCUGGCGGCGAAGUAGACGGUCGGGUCCA
GAUGCACGAGGAAGUACGCCGUGUCAAGCGUUGAGACACGGUGUACUACUAUCGUUCGCGAUAGUCGCAAAGGAUUCGUCCUUUGGUCAAUGUUCGCAUUG
ACACCGUGUUUUACCCGGACACCACCGGGCAGACAAUGAAGCGCGGUCGUCCUCGUGCUUCUGUCGAGUAGAGUGUGGGCUGCUUUGUUGCCACAGUUUCC
GUAACAAUGUCUGAGUUACGGAGACUGGUUACUCUUCGGAGUAACGCCGAGAUUCC

Table S2. PCR primers and RNA keys.

Forward primer	CAGACTTCTTACGTGATCCATCCG
Reverse primer	GGAATCTCGGCGTTACTCCG
Key A	GCGUUGCAUGGUGUAAU
Key B	UGUCUGCCCGGUGGUGU
Key C (negative control)	AACCUAACUCAAUCUCU
Key D (negative control)	UAACAAAACAGACAAAG
Key A-t	GCGUUGCAUGGUGUAAU-ACGUCG
Key B-t	UGUCUGCCCGGUGGUGU-UGAAGC
Key t-A	ACGUCG-GCGUUGCAUGGUGUAAU
Key t-B	UGAAGC-UGUCUGCCCGGUGGUGU
Anti-key A-t*	CGACGU-AUUACACCAUGCAACGC
Anti-key B-t*	GCUUCA-ACACCACCGGGCAGACA
Anti-key E (negative control)	CUGCACAGAACGGGAUUCUUUCA
Anti-key F (negative control)	GGGAAGGUGUUUGCUGGGAGUGA
